# Supplementary material for: Anticancer Activity of Chalcones and Its Derivatives: Review and In Silico Studies
Source: Molecules. 2023 May 10;28(10):4009. doi: 10.3390/molecules28104009 (PMC10223217; doi:10.3390/molecules28104009)
Supplement: Supplementary file 1 [file molecules-28-04009-s001.zip › molecules-2334237-supplementary.pdf]

molecules

MDPI

Article

Anticancer Activity of Chalcones and Its Derivatives: Review and In Silico Studies

Fernando Ferreira Leite, Natália Ferreira de Sousa, Bruno Hanrry Melo de Oliveira, Gabrielly Diniz Duarte, Maria Denise Leite Ferreira, Marcus Tullius Scotti, José Maria Barbosa Filho, Luís Cezar Rodrigues, Ricardo Olímpio de Moura, Francisco Jaime Bezerra Mendonça-Junior and Luciana Scotti

Supplementary Material

Tabela S1: Anticancer activity of chalcone-like compounds for various types of cancer with their respective cell lineage.

ID	Smiles	Activity	Unit	Assessment Method	Cell Lineage
**1.**	COC1=CC(OC)=C(C(=O)\C=C\C2=CC=CC=C2)C(O)=C1	25	µM	IC50	LoVo
**2.**	COC1=CC2=C(C(=O)CC(O2)C2=CC=CC=C2)C(OC)=C1	50	µM	IC50	LoVo
**3.**	CC1=NC(C)=C(C)N=C1\C=C\C(=O)C1=CC=CO1	7,9	µM	IC50	A375
**4.**	COC1=CC=C2OC(=CC2=C1)C(=O)\C=C\C1=C(C)N=C(C)C(C)=N1	5,16	µM	IC50	A375
**5.**	CC1=CSC(=C1)C1=CC=C(C=C1)C(=O)\C=C\C1=NC(C)=C(C)N=C1C	3,68	µM	IC50	A375
**6.**	CCOC1=CC=C(C=C1)C(=O)\C=C\C1=CC=C(Cl)C=C1	49,17	µM	IC50	A-375
**7.**	CCOC1=CC=C(C=C1)C(=O)\C=C\C1=CC=C(Br)C=C1	40,76	µM	ic50	A-375
**8.**	CCOC1=CC=C(C=C1)C(=O)\C=C\C1=CC=C(C=C1)N(O)O	76,01	µM	ic50	A-375
**9.**	CCOC1=CC=C(C=C1)C(=O)\C=C\C1=CC=C(OC)C=C1	71,19	µM	ic50	A-375
**10.**	CCOC1=CC=C(C=C1)C(=O)\C=C\C1=CC=C(OC)C(OC)=C1	47,06	µM	ic50	A-375
**11.**	O=C(\C=C\C1=CC=C(OCCCCOC2=CC=C(\C=C\C(=O)C3=CC=CO3)C=C2) C=C1)C1=CC=CO1	26,1	µM	IC50	A431

**12.**	O=C(\C=C\C1=CC=C(OCCCCOC2=CC=C(\C=C\C(=O)C3=CC=CO3)C=C2)C=C1)C1=CC=CO1	14,4	µM	IC50	A431
**13.**	[H]N1C2CCCCC2N([H])[Pt]11(Cl)(OC(=O)CCCC(=O)OC2=C(OC)C=CC(\C=C\C(=O)C3=CC(OC)=C(OC)C(OC)=C3)=C2)OC(=O)C(=O)O1	0,39	µM	IC50	A549
**14.**	COC1=CC=C(C=C1)C1ON=C2C1SC(N2C1=CC=C(Cl)C=C1)C1=CC(CC2=CC(C3SC4C(ON=C4N3C3=CC=C(Cl)C=C3)C3=CC=C(OC)C=C3)=C(OC)C=C2)=CC=C1OC	35,29	µg/mL	IC50	A549
**15.**	CC1=CC=C(C=C1)S(=O)(=O)NC1=CC=C(\C=C\C2=CC=C(F)C=C2)C=C1C(=O)\C=C\C1=CC=C(F)C=C1	5,4	µM	IC50	A549
**16.**	COC1=CC=C(\C=C\C2=CC=C(NS(=O)(=O)C3=CC=C(C)C=C3)C(=C2)C(=O)\C=C\C2=CC=C(F)C=C2)C=C1	50,3	µM	IC50	A549
**17.**	CC(C)C(NC1=NC(NC2=CC=C(C=C2)C(=O)\C=C\C2=CC=C(Cl)C=C2)=NC(NC(C(C)C)C(O)=O)=N1)C(O)=O	24,5	µM	IC50	A549
**18.**	OC(=O)C(CC1=CC=CC=C1)NC1=NC(NC2=CC=C(C=C2)C(=O)\C=C\C2=CC=C(Cl)C=C2)=NC(NC(CC2=CC=CC=C2)C(O)=O)=N1	17	µM	IC50	A549
**19.**	CC1=C(C(=O)\C=C\C2=CC=CC=C2)C(=NN1C1=CC=C(C=C1)[N+]([O-])=O)C1=CC=CS1	27,7	µM	IC50	A549
**20.**	CON1C=C(C(=O)\C=C\C2=C(OC)C=C(OC)C=C2OC)C2=CC=CC=C12	< 7	µM	IC50	A549
**21.**	O=C(\C=C\C1=CC=C(OCCOC2=CC=C(\C=C\C(=O)C3=CC=CO3)C=C2)C=C1)C1=CC=CO1	24,9	µM	IC50	A549
**22.**	[H]C1=CC(OC)=C([H])C=C1C1CC(=NN1C(C)=O)C1=C([H])C2=C(OCO2)C([H])=C1OC	4.32 a 0.28	µM	IC50	A-549
**23.**	[H]C1=CC2=C(OCO2)C=C1C1CC(=NN1C(C)=O)C1=C([H])C(OC)=C(OC)C(OC)=C1[H]	>100	µM	IC50	A-549
**24.**	[H]C1=CC2=C(OCO2)C=C1C1CC(=NN1C(C)=O)C1=C([H])C([H])=C(C)C([H])=C1[H]	>100	µM	IC50	A-549
**25.**	[H]C1=CC2=C(OCO2)C=C1C1CC(=NN1C(C)=O)C1=C([H])C([H])=C([H])C([H])=C1OC	>100	µM	IC50	A-549

**26.**	[H]C1=CC2=C(OCO2)C=C1C1CC(=NN1C(C)=O)C1=C([H])C([H])=C2OCOC2=C1[H]	60.89 a 4.30	µM	IC50	A-549
**27.**	[H]C1=CC2=C(OCO2)C=C1C1CC(=NN1C(C)=O)C1=C([H])C([H])=C(OC(F)(F)F)C([H])=C1[H]	21.46 a 4.26	µM	IC50	A-549
**28.**	[H]C1=CC2=C(OCO2)C=C1C1CC(=NN1C(C)=O)C1=C([H])C([H])=C(OC)C([H])=C1[H]	>100	µM	IC50	A-549
**29.**	[H]C1=C([H])C(=C([H])C([H])=C1OC)C1=NN(C(C1)C1=CC2=C(OCO2)C=C1OC)C(C)=O	>100	µM	IC50	A-549
**30.**	[H]C1=CC(O)=C([H])C=C1C1CC(=NN1C(C)=O)C1=C(OC)C([H])=C2OCSC2=C1[H]	79.46 a 4.97	µM	IC50	A-549
**31.**	CC1=NC(C)=C(C)N=C1\C=C\C(=O)C1=CC=CO1	5,11	µM	IC50	A-549
**32.**	COC1=CC=C2OC(=CC2=C1)C(=O)\C=C\C1=C(C)N=C(C)C(C)=N1	5,77	µM	IC50	A-549
**33.**	CC1=CSC(=C1)C1=CC=C(C=C1)C(=O)\C=C\C1=NC(C)=C(C)N=C1C	5,39	µM	IC50	A-549
**34.**	COC1=CC(OC)=C(C(=O)\C=C\C2=CC=CC=C2)C(O)=C1	11	µg/ml	IC50	A549
**35.**	[H]C1=C([H])C(=CC=C1\C=C\C(=C)C1=C(C)N(N=N1)C1=CC=C(Br)C=C1)N(C)C	98,13	µM	IC50	A549/ATCC
**36.**	[H]C1=C(OC)C(OC)=CC=C1\C=C\C(=C)C1=C(C)N(N=N1)C1=CC=C(Br)C=C1	73,63	µM	IC50	A549/ATCC
**37.**	[H]C1=C([H])C(\C=C\C(=O)C2=C(C)N(N=N2)C2=CC=C(Br)C=C2)=CC=C1OC	101,75	µM	IC50	A549/ATCC
**38.**	[H]C1=CC=C(\C=C\C(=O)C2=C(C)N(N=N2)C2=CC=C(Br)C=C2)C(OC)=C1[H]	85,19	µM	IC50	A549/ATCC
**39.**	[H]C1=CC=C(\C=C\C(=O)C2=C(C)N(N=N2)C2=CC=C(Br)C=C2)C([H])=C1[N+]([O-])=O	91,07	µM	IC50	A549/ATCC
**40.**	[H]C1=C([H])C(\C=C\C(=O)C2=C(C)N(N=N2)C2=CC=C(Br)C=C2)=CC=C1Cl	101,83	µM	IC50	A549/ATCC
**41.**	[H]C1=C([H])C(=CC=C1\C=C\C(=O)C1=C(C)N(N=N1)C1=CC=C(F)C=C1)N(C)C	95,66	µM	IC50	A549/ATCC

**42.**	[H]C1=C(OC)C(OC)=CC=C1\C=C\C(=O)C1=C(C)N(N=N1)C1=CC=C(F)C=C1	85,95	μM	IC50	A549/ATCC
**43.**	[H]C1=C([H])C(\C=C\C(=O)C2=C(C)N(N=N2)C2=CC=C(F)C=C2)=CC=C1OC	93	μM	IC50	A549/ATCC
**44.**	[H]C1=CC=C(\C=C\C(=O)C2=C(C)N(N=N2)C2=CC=C(F)C=C2)C(OC)=C1[H]	41,66	μM	IC50	A549/ATCC
**45.**	[H]C1=C([H])C(=CC=C1\C=C\C(=O)C1=C(C)N(N=N1)C1=CC=C(C=C1)C(O)=O)N(C)C	95,7	μM	IC50	A549/ATCC
**46.**	[H]C1=C(OC)C(OC)=CC=C1\C=C\C(=O)C1=C(C)N(N=N1)C1=CC=C(C=C1)C(O)=O	97,55	μM	IC50	A549/ATCC
**47.**	[H]C1=C([H])C(\C=C\C(=O)C2=C(C)N(N=N2)C2=CC=C(C=C2)C(O)=O)=CC=C1OC	100,93	μM	IC50	A549/ATCC
**48.**	[H]C1=CC=C(\C=C\C(=O)C2=C(C)N(N=N2)C2=CC=C(C=C2)C(O)=O)C(OC)=C1[H]	90,31	μM	IC50	A549/ATCC
**49.**	COC1=CC(OC)=C(C(=O)\C=C\C2=C(OC)C=CC=C2OC)C(OC)=C1	0,17	μM	IC50	ABCG2
**50.**	COC1=CC=C(\C=C\C(=O)C2=CC=C(NC3=C4C=C(OC)C(OC)=CC4=NC=N3)C=C2)C=C1OC	0,19	μM	IC50	ABCG2
**51.**	COC1=CC=C(C=C1OC)C(=O)\C=C\C1=CN(C)C2=CC=C(C=C12)C(=O)C1=CC(OC)=C(OC)C(OC)=C1	0,96	μM	GI50	AW13516
**52.**	COC1=CC(\C=C\C(=O)C2=CC=C3OC(=O)N(CN4CCCCC4)C3=C2)=CC(OC)=C1OC	3,6	μM	IC50	BV-173
**53.**	COC1=CC(\C=C\C(=O)C2=CC=C3OC(=O)N(CN4CCOCC4)C3=C2)=CC(OC)=C1OC	6,4	μM	IC50	BV-173
**54.**	COC1=CC(\C=C\C(=O)C2=CC=C3OC(=O)N(CN4CCSCC4)C3=C2)=CC(OC)=C1OC	5,1	μM	IC50	BV-173
**55.**	COC1=CC(\C=C\C(=O)C2=CC=C3OC(=O)N(CN4CCN(CC5=CC=CC=C5)CC4)C3=C2)=CC(OC)=C1OC	5,5	μM	IC50	BV-173

**56.**	COC1=CC(\C=C\C(=O)C2=CC=C3OC(=O)N(CN4CCN(CC4)C4=C(OC)C=CC=C4)C3=C2)=CC(OC)=C1OC	5,9	μM	IC50	BV-173
**57.**	O=C(\C=C\C1=COC2=CC=CC=C2C1=O)C1=CC2=C(OC1=O)C=CC=C2	2,96	μM	IC50	CAL27
**58.**	O=C(\C=C\C1=NNC=C1C1=CC2=C(OC1=O)C=CC=C2)C1=CC2=C(OC1=O)C=CC=C2	2,97	μM	IC50	CAL27
**59.**	O=C(\C=C\C1=CN(N=C1C1=CN=CC=N1)C1=CC=CC=C1)C1=CC2=CC=CC=C2OC1=O	2,82	μM	IC50	CAL27
**60.**	COC1=CC(\C=C\C(=O)C2=CC=C(Br)C=C2)=CC=C1OCCOC1=CC=C(\C=C\C(=O)C2=CC=C(Br)C=C2)C=C1OC	64,1	%	% sobrevivência	Cal51
**61.**	COC1=CC(\C=C\C(=O)C2=CC=C(N)C=C2)=CC=C1OCCOC1=CC=C(\C=C\C(=O)C2=CC=C(N)C=C2)C=C1OC	60,2	%	% sobrevivência	Cal51
**62.**	COC1=CC=C(C=C1)C(=O)\C=C\C1=CC=C(OCCOC2=CC=C(\C=C\C(=O)C3=CC=C(OC)C=C3)C=C2OC)C(OC)=C1	50,4	%	% sobrevivência	Cal51
**63.**	[O-][N+](=O)C1=CC(\C=C\C(=O)C2=CC=CC=C2)=CC=C1	8,9	μM	IC50	CCRF-CEM
**64.**	FC(F)(F)C1=CC=CC=C1\C=C\C(=O)C1=CC=CC=C1	7,5	μM	IC50	CCRF-CEM
**65.**	[O-][N+](=O)C1=CC=CC(=C1)C(=O)\C=C\C1=CC=CC=C1	6,1	μM	IC50	CCRF-CEM
**66.**	FC(F)(F)C1=CC=CC(=C1)C(=O)\C=C\C1=CC=CC=C1	11,4	μM	IC50	CCRF-CEM
**67.**	[H]C1=C([H])C(=CC=C1\C=C\C(=C)C1=C(C)N(N=N1)C1=CC=C(Br)C=C1)N(C)C	108,74	μM	IC50	Colo 205
**68.**	[H]C1=C(OC)C(OC)=CC=C1\C=C\C(=C)C1=C(C)N(N=N1)C1=CC=C(Br)C=C1	90,38	μM	IC50	Colo 205
**69.**	[H]C1=C([H])C(\C=C\C(=O)C2=C(C)N(N=N2)C2=CC=C(Br)C=C2)=CC=C1OC	110,95	μM	IC50	Colo 205
**70.**	[H]C1=CC=C(\C=C\C(=O)C2=C(C)N(N=N2)C2=CC=C(Br)C=C2)C(OC)=C1[H]	109,85	μM	IC50	Colo 205
**71.**	[H]C1=CC=C(\C=C\C(=O)C2=C(C)N(N=N2)C2=CC=C(Br)C=C2)C([H])=C1[N+]([O-])=O	106,46	μM	IC50	Colo 205

**72.**	[H]C1=C([H])C(\C=C\C(=O)C2=C(C)N(N=N2)C2=CC=C(Br)C=C2)=CC=C1Cl	106,09	µM	IC50	Colo 205
**73.**	[H]C1=C([H])C(=CC=C1\C=C\C(=O)C1=C(C)N(N=N1)C1=CC=C(F)C=C1)N(C)C	101,48	µM	IC50	Colo 205
**74.**	[H]C1=C(OC)C(OC)=CC=C1\C=C\C(=O)C1=C(C)N(N=N1)C1=CC=C(F)C=C1	106,57	µM	IC50	Colo 205
**75.**	[H]C1=C([H])C(\C=C\C(=O)C2=C(C)N(N=N2)C2=CC=C(F)C=C2)=CC=C1OC	101,97	µM	IC50	Colo 205
**76.**	[H]C1=CC=C(\C=C\C(=O)C2=C(C)N(N=N2)C2=CC=C(F)C=C2)C(OC)=C1[H]	72,37	µM	IC50	Colo 205
**77.**	[H]C1=C([H])C(=CC=C1\C=C\C(=O)C1=C(C)N(N=N1)C1=CC=C(C=C1)C(O)=O)N(C)C	105,86	µM	IC50	Colo 205
**78.**	[H]C1=C(OC)C(OC)=CC=C1\C=C\C(=O)C1=C(C)N(N=N1)C1=CC=C(C=C1)C(O)=O	108,62	µM	IC50	Colo 205
**79.**	[H]C1=C([H])C(\C=C\C(=O)C2=C(C)N(N=N2)C2=CC=C(C=C2)C(O)=O)=CC=C1OC	107,34	µM	IC50	Colo 205
**80.**	COC1=CC(O)=CC=C1C(=O)\C=C\C1=CC=C(O)C=C1	1,92	µM	IC50	DND-41
**81.**	COC1=CC=C(C=C1)C1ON=C2C1SC(N2C1=CC=C(Cl)C=C1)C1=CC(CC2=CC(C3SC4C(ON=C4N3C3=CC=C(Cl)C=C3)C3=CC=C(OC)C=C3)=C(OC)C=C2)=CC=C1OC	14,98	µg/mL	IC50	DU145
**82.**	CC(C)CC1=CC=C(C=C1)C1=NC2=C(SC(C1)C1=CC=CN1)C=CC=C2	12	µg/mL	IC50	DU-145
**83.**	CC(C)CC1=CC=C(C=C1)C1=NC2=C(SC(C1)C1=CC=CS1)C=CC=C2	18	µg/mL	IC50	DU-145
**84.**	CC(C)CC1=CC=C(C=C1)C1=NC2=C(SC(C1)C1=CC=C(Br)O1)C=CC=C2	4	µg/mL	IC50	DU-145
**85.**	NC1=NC(=CC(=N1)C1=CC=C(C=C1)[N+]([O-])=O)C1=NC=C(Cl)N=C1	112	µg/mL	IC50	DU-145
**86.**	NC1=NC(=CC(=N1)C1=CC=C(F)C=C1F)C1=NC=C(Cl)N=C1	10	µg/mL	IC50	DU-145
**87.**	NC1=NC(=CC(=N1)C1=CC=C(Cl)C=C1Cl)C1=NC=C(Cl)N=C1	12	µg/mL	IC50	DU-145
**88.**	NC1=NC(=CC(=N1)C1=NC=CC=C1)C1=NC=C(Cl)N=C1	5	µg/mL	IC50	DU-145

**89.**	[H]C1CC[C@]2([H])[C@]1([H])CC[C@]1([H])C3=C(CC[C@@]21[H])C=C1OC(=CC(=O)C1=C3)C1=CC=CC=C1	5,6	μM	IC50	DU-145
**90.**	[H]C1CC[C@]2([H])[C@]1([H])CC[C@]1([H])C3=C(CC[C@@]21[H])C=C1OC(=CC(=O)C1=C3)C1=CC=C(C)C=C1	9,7	μM	IC50	DU-145
**91.**	[H]C1CC[C@]2([H])[C@]1([H])CC[C@]1([H])C3=C(CC[C@@]21[H])C=C1OC(=CC(=O)C1=C3)C1=CC=C(Br)C=C1	6,2	μM	IC50	DU-145
**92.**	COC1=CC2=C(C=C1OC)C1N(CC2)C(=NN1C1=CC=CC=C1)C(=O)\C=C\C1=CC(OC)=C(OC)C(OC)=C1	1142	mg/kg	LD50	Ehrlich ascites carcinoma cells
**93.**	[H]C1=C([H])C(=CC=C1\C=C\C(=C)C1=C(C)N(N=N1)C1=CC=C(Br)C=C1)N(C)C	97,97	μM	IC50	EKVX
**94.**	[H]C1=C(OC)C(OC)=CC=C1\C=C\C(=C)C1=C(C)N(N=N1)C1=CC=C(Br)C=C1	62,35	μM	IC50	EKVX
**95.**	[H]C1=C([H])C(\C=C\C(=O)C2=C(C)N(N=N2)C2=CC=C(Br)C=C2)=CC=C1OC	96,89	μM	IC50	EKVX
**96.**	[H]C1=CC=C(\C=C\C(=O)C2=C(C)N(N=N2)C2=CC=C(Br)C=C2)C(OC)=C1[H]	91,62	μM	IC50	EKVX
**97.**	[H]C1=CC=C(\C=C\C(=O)C2=C(C)N(N=N2)C2=CC=C(Br)C=C2)C([H])=C1[N+]([O-])=O	92,3	μM	IC50	EKVX
**98.**	[H]C1=C([H])C(\C=C\C(=O)C2=C(C)N(N=N2)C2=CC=C(Br)C=C2)=CC=C1Cl	93,43	μM	IC50	EKVX
**99.**	[H]C1=C([H])C(=CC=C1\C=C\C(=O)C1=C(C)N(N=N1)C1=CC=C(F)C=C1)N(C)C	94,37	μM	IC50	EKVX
**100.**	[H]C1=C(OC)C(OC)=CC=C1\C=C\C(=O)C1=C(C)N(N=N1)C1=CC=C(F)C=C1	82,59	μM	IC50	EKVX
**101.**	[H]C1=C([H])C(\C=C\C(=O)C2=C(C)N(N=N2)C2=CC=C(F)C=C2)=CC=C1OC	83,29	μM	IC50	EKVX

**102.**	[H]C1=CC=C(\C=C\C(=O)C2=C(C)N(N=N2)C2=CC=C(F)C=C2)C(OC)=C1[H]	69,31	µM	IC50	EKVX
**103.**	[H]C1=C([H])C(=CC=C1\C=C\C(=O)C1=C(C)N(N=N1)C1=CC=C(C=C1)C(O)=O)N(C)C	93,83	µM	IC50	EKVX
**104.**	[H]C1=C(OC)C(OC)=CC=C1\C=C\C(=O)C1=C(C)N(N=N1)C1=CC=C(C=C1)C(O)=O	96,17	µM	IC50	EKVX
**105.**	[H]C1=C([H])C(\C=C\C(=O)C2=C(C)N(N=N2)C2=CC=C(C=C2)C(O)=O)=CC=C1OC	87,17	µM	IC50	EKVX
**106.**	[H]C1=CC=C(\C=C\C(=O)C2=C(C)N(N=N2)C2=CC=C(C=C2)C(O)=O)C(OC)=C1[H]	83,29	µM	IC50	EKVX
**107.**	COC1=CC(\C=C\C(=O)C2=CC(OC)=C(OC)C=C2)=CC(OC)=C1OC	25,54	µM	IC50	GL261
**108.**	COC1=CC=C(C=C1)C1=CC2=CC=CC=C2N=C1\C=C\C(=O)C1=CC=CC=C1	141	µM	IC50	H1299
**109.**	COC1=CC(OC)=C(C(=O)\C=C\C2=CC=CC=C2)C(O)=C1	5,1	µg/ml	IC50	H1299
**110.**	[H]C1=CC(OC)=C([H])C=C1C1CC(=NN1C(C)=O)C1=C([H])C2=C(OCO2)C([H])=C1OC	4.69 a 0.43	µM	IC50	H226
**111.**	[H]C1=CC2=C(OCO2)C=C1C1CC(=NN1C(C)=O)C1=C([H])C(OC)=C(OC)C(OC)=C1[H]	40.46 a 3.52	µM	IC50	H226
**112.**	[H]C1=CC2=C(OCO2)C=C1C1CC(=NN1C(C)=O)C1=C([H])C([H])=C(C)C([H])=C1[H]	86.70 a 0.92	µM	IC50	H226
**113.**	[H]C1=CC2=C(OCO2)C=C1C1CC(=NN1C(C)=O)C1=C([H])C([H])=C([H])C([H])=C1OC	86.70 a 4.70	µM	IC50	H226
**114.**	[H]C1=CC2=C(OCO2)C=C1C1CC(=NN1C(C)=O)C1=C([H])C([H])=C2OCOC2=C1[H]	57.99 a 2.56	µM	IC50	H226
**115.**	[H]C1=CC2=C(OCO2)C=C1C1CC(=NN1C(C)=O)C1=C([H])C([H])=C(OC(F)(F)F)C([H])=C1[H]	24.36 a 2.13	µM	IC50	H226
**116.**	[H]C1=CC2=C(OCO2)C=C1C1CC(=NN1C(C)=O)C1=C([H])C([H])=C(OC)C([H])=C1[H]	41.49 a 1.12	µM	IC50	H226

**117.**	[H]C1=C([H])C(=C([H])C([H])=C1OC)C1=NN(C(C1)C1=CC2=C(OCO2)C=C1OC)C(C)=O	61.11 a 3.12	µM	IC50	H226
**118.**	[H]C1=CC(O)=C([H])C=C1C1CC(=NN1C(C)=O)C1=C(OC)C([H])=C2OCSC2=C1[H]	38.38 a 3.53	µM	IC50	H226
**119.**	[H]C1=CC(OC)=C([H])C=C1C1CC(=NN1C(C)=O)C1=C([H])C2=C(OCO2)C([H])=C1OC	8.40 a 1.10	µM	IC50	H460
**120.**	[H]C1=CC2=C(OCO2)C=C1C1CC(=NN1C(C)=O)C1=C([H])C(OC)=C(OC)C(OC)=C1[H]	43.84 a 1.12	µM	IC50	H460
**121.**	[H]C1=CC2=C(OCO2)C=C1C1CC(=NN1C(C)=O)C1=C([H])C([H])=C(C)C([H])=C1[H]	24.53 a 1.74	µM	IC50	H460
**122.**	[H]C1=CC2=C(OCO2)C=C1C1CC(=NN1C(C)=O)C1=C([H])C([H])=C([H])C([H])=C1OC	63.43 a 3.34	µM	IC50	H460
**123.**	[H]C1=CC2=C(OCO2)C=C1C1CC(=NN1C(C)=O)C1=C([H])C([H])=C2OCOC2=C1[H]	38.84 a 1.77	µM	IC50	H460
**124.**	[H]C1=CC2=C(OCO2)C=C1C1CC(=NN1C(C)=O)C1=C([H])C([H])=C(OC(F)(F)F)C([H])=C1[H]	18.12 a 2.13	µM	IC50	H460
**125.**	[H]C1=CC2=C(OCO2)C=C1C1CC(=NN1C(C)=O)C1=C([H])C([H])=C(OC)C([H])=C1[H]	30.16 a 2.14	µM	IC50	H460
**126.**	[H]C1=C([H])C(=C([H])C([H])=C1OC)C1=NN(C(C1)C1=CC2=C(OCO2)C=C1OC)C(C)=O	30.16 a 2.14	µM	IC50	H460
**127.**	[H]C1=CC(O)=C([H])C=C1C1CC(=NN1C(C)=O)C1=C(OC)C([H])=C2OCSC2=C1[H]	43.77 a 3.97	µM	IC50	H460
**128.**	CC1=NC(C)=C(C)N=C1\C=C\C(=O)C1=CC=CO1	5,41	µM	IC50	H460
**129.**	COC1=CC=C2OC(=CC2=C1)C(=O)\C=C\C1=C(C)N=C(C)C(C)=N1	5,78	µM	IC50	H460
**130.**	CC1=CSC(=C1)C1=CC=C(C=C1)C(=O)\C=C\C1=NC(C)=C(C)N=C1C	2,72	µM	IC50	H460
**131.**	CC(C)(C)OC(=O)N1CCN(CC1)C(=S)SCC(=O)NC1=CC=C(C=C1)C(=O)\C=C\C1=CC=CC=C1F	1,1	µM	IC50	HAL-01
**132.**	COC1=C(O)C(C(=O)\C=C\C2=CC=C(O)C=C2)=C(OC)C(OC)=C1OC	33,85	µM	IC50	HCC1954

**133.**	COC1=CC(\C=C\C(=O)C2=C(OC)C(OC)=C(OC)C(OC)=C2O)=CC=C1O	25,25	μM	IC50	HCC1954
**134.**	COC1=CC=C(\C=C\C(=O)C2=C(OC)C(OC)=C(OC)C(OC)=C2O)C=C1O	9,28	μM	IC50	HCC1954
**135.**	COC1=C(O)C(O)=CC(\C=C\C(=O)C2=C(OC)C(OC)=C(OC)C(OC)=C2O)=C1	19,47	μM	IC50	HCC1954
**136.**	COC1=CC(\C=C\C(=O)C2=C(OC)C(OC)=C(OC)C(OC)=C2O)=CC(OC)=C1O C	15,98	μM	IC50	HCC1954
**137.**	[H]C1=CC=C(\C=C\C(=O)C2=C(C)N(N=N2)C2=CC=C(C=C2)C(O)=O)C(OC) =C1[H]	110,11	μM	IC50	HCC-2998
**138.**	[H]C1=C([H])C(=CC=C1\C=C\C(=C)C1=C(C)N(N=N1)C1=CC=C(Br)C=C1)N (C)C	99,87	μM	IC50	HCC-2998
**139.**	[H]C1=C(OC)C(OC)=CC=C1\C=C\C(=C)C1=C(C)N(N=N1)C1=CC=C(Br)C=C 1	83,75	μM	IC50	HCC-2998
**140.**	[H]C1=C([H])C(\C=C\C(=O)C2=C(C)N(N=N2)C2=CC=C(Br)C=C2)=CC=C1O C	106,55	μM	IC50	HCC-2998
**141.**	[H]C1=CC=C(\C=C\C(=O)C2=C(C)N(N=N2)C2=CC=C(Br)C=C2)C(OC)=C1[H]	93,9	μM	IC50	HCC-2998
**142.**	[H]C1=CC=C(\C=C\C(=O)C2=C(C)N(N=N2)C2=CC=C(Br)C=C2)C([H])=C1[N+]([O-])=O	101,24	μM	IC50	HCC-2998
**143.**	[H]C1=C([H])C(\C=C\C(=O)C2=C(C)N(N=N2)C2=CC=C(Br)C=C2)=CC=C1C l	95,43	μM	IC50	HCC-2998
**144.**	[H]C1=C([H])C(=CC=C1\C=C\C(=O)C1=C(C)N(N=N1)C1=CC=C(F)C=C1)N(C)C	102,9	μM	IC50	HCC-2998
**145.**	[H]C1=C(OC)C(OC)=CC=C1\C=C\C(=O)C1=C(C)N(N=N1)C1=CC=C(F)C=C 1	102,85	μM	IC50	HCC-2998
**146.**	[H]C1=C([H])C(\C=C\C(=O)C2=C(C)N(N=N2)C2=CC=C(F)C=C2)=CC=C1O C	98,77	μM	IC50	HCC-2998
**147.**	[H]C1=CC=C(\C=C\C(=O)C2=C(C)N(N=N2)C2=CC=C(F)C=C2)C(OC)=C1[H]	81,31	μM	IC50	HCC-2998

**148.**	[H]C1=C([H])C(=CC=C1\C=C\C(=O)C1=C(C)N(N=N1)C1=CC=C(C=C1)C(O)=O)N(C)C	98,69	µM	IC50	HCC-2998
**149.**	[H]C1=C(OC)C(OC)=CC=C1\C=C\C(=O)C1=C(C)N(N=N1)C1=CC=C(C=C1)C(O)=O	105,62	µM	IC50	HCC-2998
**150.**	[H]C1=C([H])C(\C=C\C(=O)C2=C(C)N(N=N2)C2=CC=C(C=C2)C(O)=O)=CC=C1OC	99,27	µM	IC50	HCC-2998
**151.**	[H]C1=CC=C(\C=C\C(=O)C2=C(C)N(N=N2)C2=CC=C(C=C2)C(O)=O)C(OC)=C1[H]	102,41	µM	IC50	HCC-2998
**152.**	O=C(CC1=CC=C(\C=C\C(=O)C2=NC=CN2)C=C1)NC1=CC=CC=C1	45,33	10 µM	% crescimento	HCT116
**153.**	O=C(CC1=CC=C(\C=C\C(=O)C2=NC=CN2)C=C1)NC1CCCCC1	70,02	10 µM	% crescimento	HCT116
**154.**	CC1=CC=C(NC(=O)CC2=CC=C(\C=C\C(=O)C3=NC=CN3)C=C2)C=C1	41,34	10 µM	% crescimento	HCT116
**155.**	COC1=CC=C(NC(=O)CC2=CC=C(\C=C\C(=O)C3=NC=CN3)C=C2)C=C1	66,84	10 µM	% crescimento	HCT116
**156.**	CN(C)C1=CC=C(NC(=O)CC2=CC=C(\C=C\C(=O)C3=NC=CN3)C=C2)C=C1	52,06	10 µM	% crescimento	HCT116
**157.**	O=C(CC1=CC=C(\C=C\C(=O)C2=NC=CN2)C=C1)NC1=C2C=CC=CC2=CC=C1	76,31	10 µM	% crescimento	HCT116
**158.**	FC1=CC=C(NC(=O)CC2=CC=C(\C=C\C(=O)C3=NC=CN3)C=C2)C=C1	27,48	10 µM	% crescimento	HCT116
**159.**	ClC1=CC=C(NC(=O)CC2=CC=C(\C=C\C(=O)C3=NC=CN3)C=C2)C=C1	21,1	10 µM	% crescimento	HCT116
**160.**	BrC1=CC=C(NC(=O)CC2=CC=C(\C=C\C(=O)C3=NC=CN3)C=C2)C=C1	17,88	10 µM	% crescimento	HCT116
**161.**	FC(F)(F)C1=CC=C(NC(=O)CC2=CC=C(\C=C\C(=O)C3=NC=CN3)C=C2)C=C1	23,48	10 µM	% crescimento	HCT116
**162.**	FC1=CC(NC(=O)CC2=CC=C(\C=C\C(=O)C3=NC=CN3)C=C2)=CC(F)=C1F	51,1	10 µM	% crescimento	HCT116
**163.**	FC1=C(F)C(F)=C(NC(=O)CC2=CC=C(\C=C\C(=O)C3=NC=CN3)C=C2)C(F)=C1F	91,77	10 µM	% crescimento	HCT116
**164.**	CN1C=CN=C1C(=O)\C=C\C1=CC=C(CC(=O)NC2CCCCC2)C=C1	57,67	10 µM	% crescimento	HCT116
**165.**	CN1C=CN=C1C(=O)\C=C\C1=CC=C(CC(=O)NC2=CC=CC=C2)C=C1	15,9	10 µM	% crescimento	HCT116
**166.**	CN1C=CN=C1C(=O)\C=C\C1=CC=C(CC(=O)NC2=CC=C(Cl)C=C2)C=C1	19,33	10 µM	% crescimento	HCT116
**167.**	CN1C=CN=C1C(=O)\C=C\C1=CC=C(CC(=O)NC2=CC=C(Br)C=C2)C=C1	19,91	10 µM	% crescimento	HCT116
**168.**	CN1C=CN=C1C(=O)\C=C\C1=CC=C(CC(=O)NC2=CC=C(F)C=C2)C=C1	33,11	10 µM	% crescimento	HCT116

**169.**	FC1=CC=C(NC(=O)CC2=CC=C(\C=C\C(=O)C3=NC=CN3CC3=CC=CC=C3)C=C2)C=C1	54,69	10 μM	% crescimento	HCT116
**170.**	CCN1C=CN=C1C(=O)\C=C\C1=CC=C(CC(=O)NC2=CC=C(F)C=C2)C=C1	29,2	10 μM	% crescimento	HCT116
**171.**	CN1C=CN=C1C(=O)\C=C\C1=CC=C(CC(=O)NC2=CC=C(C=C2)C(F)(F)F)C=C1	12,97	10 μM	% crescimento	HCT116
**172.**	CN1C=CN=C1C(=O)\C=C\C1=CC=C(CC(=O)NC2=CC(F)=C(F)C(F)=C2)C=C1	22,66	10 μM	% crescimento	HCT116
**173.**	CN1C=CN=C1C(=O)\C=C\C1=CC=C(CC(=O)NC2=C(F)C(F)=C(F)C(F)=C2F)C=C1	61,43	10 μM	% crescimento	HCT116
**174.**	CN1C=CN=C1C(=O)\C=C\C1=CC=C(CC(=O)NC2=CC(=CC(=C2)C(F)(F)F)C(F)(F)F)C=C1	68,98	10 μM	% crescimento	HCT116
**175.**	CN1C=CN=C1C(=O)\C=C\C1=CC=C(CC(=O)NC2=CC=C(C=C2)C#C)C=C1	53,49	10 μM	% crescimento	HCT116
**176.**	O=C(CC1=CC=C(CCC(=O)C2=NC=CN2)C=C1)NC1=CC=CC=C1	92,81	10 μM	% crescimento	HCT116
**177.**	CN1C=CN=C1C(=O)C1CC1C1=CC=C(CC(=O)NC2=CC=C(Br)C=C2)C=C1	98,67	10 μM	% crescimento	HCT116
**178.**	O=C(CC1=CC=C(\C=C\C(=O)C2=NC=CS2)C=C1)NC1=CC=CC=C1	84,29	10 μM	% crescimento	HCT116
**179.**	O=C(CC1=CC=C(\C=C\C(=O)C2=NC=CC=N2)C=C1)NC1=CC=CC=C1	99,14	10 μM	% crescimento	HCT116
**180.**	OC1=CC=CC=C1C(=O)\C=C\C1=CC=C(CC(=O)NC2=CC=CC=C2)C=C1	85,87	10 μM	% crescimento	HCT116
**181.**	OC(CC(=O)C1=CC=CC=C1O)C1=CC=C(CC(=O)NC2=CC=CC=C2)C=C1	91,98	10 μM	% crescimento	HCT116
**182.**	CN(C)C1=CC=C(NC(=O)CC2=CC=C(\C=C\C(=O)C3=CC=CC=C3O)C=C2)C=C1	89,56	10 μM	% crescimento	HCT116
**183.**	OC1=CC=CC=C1C(=O)\C=C\C1=CC=C(CC(=O)NC2=C3C=CC=CC3=CC=C2)C=C1	85,87	10 μM	% crescimento	HCT116
**184.**	[H]C1=CC(\C=C\C(=O)C2=CC=C(C=C2)N2C=C(CN3CCN(CC3)C3=CC4=C(C=C3F)C(=O)C(=CN4C3CC3)C(O)=O)N=N2)=CC([H])=C1[H]	3,57	μM	IC50	HCT116
**185.**	[H]C1=C([H])C(Cl)=CC(\C=C\C(=O)C2=CC=C(C=C2)N2C=C(CN3CCN(CC3)C3=CC4=C(C=C3F)C(=O)C(=CN4C3CC3)C(O)=O)N=N2)=C1	4,81	μM	IC50	HCT116
**186.**	[H]C1=CC(\C=C\C(=O)C2=CC=C(C=C2)N2C=C(CN3CCN(CC3)C3=CC4=C(C=C3F)C(=O)C(=CN4C3CC3)C(O)=O)N=N2)=CC([H])=C1F	4,32	μM	IC50	HCT116

**187.**	[H]C1=C(OC)C(OC)=CC(\C=C\C(=O)C2=CC=C(C=C2)N2C=C(CN3CCN(CC3)C3=CC4=C(C=C3F)C(=O)C(=CN4C3CC3)C(O)=O)N=N2)=C1	4,87	µM	IC50	HCT116
**188.**	COC1=CC(\C=C\C(=O)C2=CC=C(C=C2)N2C=C(CN3CCN(CC3)C3=CC4=C(C=C3F)C(=O)C(=CN4C3CC3)C(O)=O)N=N2)=CC(OC)=C1OC	2,53	µM	IC50	HCT116
**189.**	COC1=CC(OC2=CC(OC)=C(OC)C(OC)=C2)=C(C(/C=C/C3=CC=C(OC)C=C3)=O)C=C1	6,31	µM	IC50	HCT116
**190.**	CON1C=C(C(=O)\C=C\C2=C(OC)C=C(OC)C=C2OC)C2=CC=CC=C12	< 7	µM	IC50	HCT116
**191.**	CON1C=C(C(=O)\C=C\C2=C(OC)C=CC=C2OC)C2=CC=CC=C12	< 7	µM	IC50	HCT116
**192.**	CON1C=C(C(=O)\C=C\C2=CC(OC)=C(OC)C(OC)=C2)C2=CC=CC=C12	< 7	µM	IC50	HCT116
**193.**	O=C(\C=C\C1=CC=C(OCCOC2=CC=C(\C=C\C(=O)C3=CC=CO3)C=C2)C=C1)C1=CC=CO1	13,7	µM	IC50	HCT116
**194.**	CN(C)C1=CC=C(\C=N\C2=NC(=CC(=N2)C2=CC=C(C=C2)C2=NC(\N=C\C3=CC=C(C=C3)N(C)C)=NC(=C2)C2=CC=C(Br)C=C2)C2=CC=C(Br)C=C2)C=C1	0,01	µM	IC50	HCT116
**195.**	ClC1=CC(Cl)=C(\C=N\C2=NC(=CC(=N2)C2=CC=C(C=C2)C2=NC(\N=C\C3=CC=C(Cl)C=C3Cl)=NC(=C2)C2=CC=C(Br)C=C2)C2=CC=C(Br)C=C2)C=C1	0,02	µM	IC50	HCT116
**196.**	[H]C1=C([H])C(=CC=C1\C=C\C(=C)C1=C(C)N(N=N1)C1=CC=C(Br)C=C1)N(C)C	84,83	µM	IC50	HCT-116
**197.**	[H]C1=C(OC)C(OC)=CC=C1\C=C\C(=C)C1=C(C)N(N=N1)C1=CC=C(Br)C=C1	5,41	µM	IC50	HCT-116
**198.**	[H]C1=C([H])C(\C=C\C(=O)C2=C(C)N(N=N2)C2=CC=C(Br)C=C2)=CC=C1OC	97,89	µM	IC50	HCT-116
**199.**	[H]C1=CC=C(\C=C\C(=O)C2=C(C)N(N=N2)C2=CC=C(Br)C=C2)C(OC)=C1[H]	66,23	µM	IC50	HCT-116
**200.**	[H]C1=CC=C(\C=C\C(=O)C2=C(C)N(N=N2)C2=CC=C(Br)C=C2)C([H])=C1[N+]([O-])=O	52,16	µM	IC50	HCT-116
**201.**	[H]C1=C([H])C(\C=C\C(=O)C2=C(C)N(N=N2)C2=CC=C(Br)C=C2)=CC=C1Cl	84,46	µM	IC50	HCT-116

**202.**	[H]C1=C([H])C(=CC=C1\C=C\C(=O)C1=C(C)N(N=N1)C1=CC=C(F)C=C1)N(C)C	96,53	μM	IC50	HCT-116
**203.**	[H]C1=C(OC)C(OC)=CC=C1\C=C\C(=O)C1=C(C)N(N=N1)C1=CC=C(F)C=C1	64,06	μM	IC50	HCT-116
**204.**	[H]C1=C([H])C(\C=C\C(=O)C2=C(C)N(N=N2)C2=CC=C(F)C=C2)=CC=C1OC	82,6	μM	IC50	HCT-116
**205.**	[H]C1=CC=C(\C=C\C(=O)C2=C(C)N(N=N2)C2=CC=C(F)C=C2)C(OC)=C1[H]	10,68	μM	IC50	HCT-116
**206.**	[H]C1=C([H])C(=CC=C1\C=C\C(=O)C1=C(C)N(N=N1)C1=CC=C(C=C1)C(O)=O)N(C)C	94,09	μM	IC50	HCT-116
**207.**	[H]C1=C(OC)C(OC)=CC=C1\C=C\C(=O)C1=C(C)N(N=N1)C1=CC=C(C=C1)C(O)=O	91,62	μM	IC50	HCT-116
**208.**	[H]C1=C([H])C(\C=C\C(=O)C2=C(C)N(N=N2)C2=CC=C(C=C2)C(O)=O)=CC=C1OC	87,37	μM	IC50	HCT-116
**209.**	[H]C1=CC=C(\C=C\C(=O)C2=C(C)N(N=N2)C2=CC=C(C=C2)C(O)=O)C(OC)=C1[H]	67,98	μM	IC50	HCT-116
**210.**	COC1=CC=C(\C=C\C(=O)CC(C)C)C=C1OC	6.85 ± 0.71	μM	IC50	HCT-116
**211.**	COC1=CC(\C=C\C(=O)CC(C)C)=CC=C1OCC1=CC=CC=C1	7.9 ± 1.37	μM	IC50	HCT-116
**212.**	CCCCN1C(=O)C(\C=C\C(=O)\C=C\C2=CC3=C(C=CC=C3)N(CCCC)C2=O)=CC2=C1C=CC=C2	0,16	μM	GI50	HCT-116
**213.**	[H]C1=C([H])C(=CC=C1\C=C\C(=C)C1=C(C)N(N=N1)C1=CC=C(Br)C=C1)N(C)C	91,53	μM	IC50	HCT-15
**214.**	[H]C1=C(OC)C(OC)=CC=C1\C=C\C(=C)C1=C(C)N(N=N1)C1=CC=C(Br)C=C1	60,73	μM	IC50	HCT-15
**215.**	[H]C1=C([H])C(\C=C\C(=O)C2=C(C)N(N=N2)C2=CC=C(Br)C=C2)=CC=C1OC	96,87	μM	IC50	HCT-15
**216.**	[H]C1=CC=C(\C=C\C(=O)C2=C(C)N(N=N2)C2=CC=C(Br)C=C2)C(OC)=C1[H]	73,51	μM	IC50	HCT-15

217.	[H]C1=CC=C(\C=C\C(=O)C2=C(C)N(N=N2)C2=CC=C(Br)C=C2)C([H])=C1[N+]([O-])=O	81,47	µM	IC50	HCT-15
218.	[H]C1=C([H])C(\C=C\C(=O)C2=C(C)N(N=N2)C2=CC=C(Br)C=C2)=CC=C1Cl	92,13	µM	IC50	HCT-15
219.	[H]C1=C([H])C(=CC=C1\C=C\C(=O)C1=C(C)N(N=N1)C1=CC=C(F)C=C1)N(C)C	90,67	µM	IC50	HCT-15
220.	[H]C1=C(OC)C(OC)=CC=C1\C=C\C(=O)C1=C(C)N(N=N1)C1=CC=C(F)C=C1	63,7	µM	IC50	HCT-15
221.	[H]C1=C([H])C(\C=C\C(=O)C2=C(C)N(N=N2)C2=CC=C(F)C=C2)=CC=C1OC	79,72	µM	IC50	HCT-15
222.	[H]C1=CC=C(\C=C\C(=O)C2=C(C)N(N=N2)C2=CC=C(F)C=C2)C(OC)=C1[H]	22,93	µM	IC50	HCT-15
223.	[H]C1=C([H])C(=CC=C1\C=C\C(=O)C1=C(C)N(N=N1)C1=CC=C(C=C1)C(O)=O)N(C)C	95,33	µM	IC50	HCT-15
224.	[H]C1=C(OC)C(OC)=CC=C1\C=C\C(=O)C1=C(C)N(N=N1)C1=CC=C(C=C1)C(O)=O	89,58	µM	IC50	HCT-15
225.	[H]C1=C([H])C(\C=C\C(=O)C2=C(C)N(N=N2)C2=CC=C(C=C2)C(O)=O)=CC=C1OC	105,12	µM	IC50	HCT-15
226.	[H]C1=CC=C(\C=C\C(=O)C2=C(C)N(N=N2)C2=CC=C(C=C2)C(O)=O)C(OC)=C1[H]	71,57	µM	IC50	HCT-15
227.	COC1=CC(\C=C\C(=O)C2=CC=C3OC(=O)N(CN4CCCCC4)C3=C2)=CC(OC)=C1OC	20,5	µM	IC50	HD-MY-Z
228.	COC1=CC(\C=C\C(=O)C2=CC=C3OC(=O)N(CN4CCOCC4)C3=C2)=CC(OC)=C1OC	48,2	µM	IC50	HD-MY-Z
229.	COC1=CC(\C=C\C(=O)C2=CC=C3OC(=O)N(CN4CCSCC4)C3=C2)=CC(OC)=C1OC	14,8	µM	IC50	HD-MY-Z
230.	COC1=CC(\C=C\C(=O)C2=CC=C3OC(=O)N(CN4CCN(CC5=CC=CC=C5)CC4)C3=C2)=CC(OC)=C1OC	21,4	µM	IC50	HD-MY-Z

**231.**	COC1=CC(\C=C\C(=O)C2=CC=C3OC(=O)N(CN4CCN(CC4)C4=C(OC)C=CC=C4)C3=C2)=CC(OC)=C1OC	20	μM	IC50	HD-MY-Z
**232.**	[H]C1=CC(OC)=C([H])C=C1C1CC(=NN1C(C)=O)C1=C([H])C2=C(OCO2)C([H])=C1OC	20.95 a 2.34	μM	IC50	HEK293
**233.**	[H]C1=CC2=C(OCO2)C=C1C1CC(=NN1C(C)=O)C1=C([H])C(OC)=C(OC)C(OC)=C1[H]	> 100	μM	IC50	HEK293
**234.**	[H]C1=CC2=C(OCO2)C=C1C1CC(=NN1C(C)=O)C1=C([H])C([H])=C(C)C([H])=C1[H]	> 100	μM	IC50	HEK293
**235.**	[H]C1=CC2=C(OCO2)C=C1C1CC(=NN1C(C)=O)C1=C([H])C([H])=C([H])C([H])=C1OC	> 100	μM	IC50	HEK293
**236.**	[H]C1=CC2=C(OCO2)C=C1C1CC(=NN1C(C)=O)C1=C([H])C([H])=C2OCOC2=C1[H]	46.99 a 3.18	μM	IC50	HEK293
**237.**	[H]C1=CC2=C(OCO2)C=C1C1CC(=NN1C(C)=O)C1=C([H])C([H])=C(OC(F)(F)F)C([H])=C1[H]	42.99 a 1.23	μM	IC50	HEK293
**238.**	[H]C1=CC2=C(OCO2)C=C1C1CC(=NN1C(C)=O)C1=C([H])C([H])=C(OC)C([H])=C1[H]	> 100	μM	IC50	HEK293
**239.**	[H]C1=C([H])C(=C([H])C([H])=C1OC)C1=NN(C(C1)C1=CC2=C(OCO2)C=C1OC)C(C)=O	> 100	μM	IC50	HEK293
**240.**	[H]C1=CC(O)=C([H])C=C1C1CC(=NN1C(C)=O)C1=C(OC)C([H])=C2OCSC2=C1[H]	34.43 a 2.01	μM	IC50	HEK293
**241.**	C1C(NN=C1C1=C2C=CC=CC2=CC2=C1C=CC=C2)C1=CC=CC=C1	0,22	μM	IC50	HeLa
**242.**	ClC1=CC=C(C=C1)C1CC(=NN1)C1=C2C=CC=CC2=CC2=C1C=CC=C2	25	μM	IC50	HeLa
**243.**	FC1=CC=C(C=C1)C1CC(=NN1)C1=C2C=CC=CC2=CC2=C1C=CC=C2	29,56	μM	IC50	HeLa
**244.**	CC1=CC=C(C=C1)C1CC(=NN1)C1=C2C=CC=CC2=CC2=C1C=CC=C2	2,03	μM	IC50	HeLa
**245.**	ClC1=CC(=CC=C1)C1CC(=NN1)C1=C2C=CC=CC2=CC2=C1C=CC=C2	2,58	μM	IC50	HeLa
**246.**	[O-][N+](=O)C1=CC=C(C=C1)C1CC(=NN1)C1=C2C=CC=CC2=CC2=C1C=CC=C2	27,29	μM	IC50	HeLa
**247.**	BrC1=CC=C(C=C1)C1CC(=NN1)C1=C2C=CC=CC2=CC2=C1C=CC=C2	1,96	μM	IC50	HeLa

**248.**	FC1=CC(=CC=C1)C1CC(=NN1)C1=C2C=CC=CC2=CC2=C1C=CC=C2	1,1	μM	IC50	HeLa
**249.**	[O-][N+](=O)C1=CC(=CC=C1)C1CC(=NN1)C1=C2C=CC=CC2=CC2=C1C=CC=C2	1,3	μM	IC50	HeLa
**250.**	CN(C)C1=CC=C(C=C1)C1CC(=NN1)C1=C2C=CC=CC2=CC2=C1C=CC=C2	0,3	μM	IC50	HeLa
**251.**	C1C(NN=C1C1=C2C=CC=CC2=CC2=C1C=CC=C2)C1=CC=C(C=C1)C1=CC=CO1	1,88	μM	IC50	HeLa
**252.**	C1C(NN=C1C1=C2C=CC=CC2=CC2=C1C=CC=C2)C1=CC=CO1	0,3	μM	IC50	HeLa
**253.**	COC1=CC(=CC(OC)=C1OC)C1CC(=NN1)C1=C2C=CC=CC2=CC2=C1C=CC=C2	0,1	μM	IC50	HeLa
**254.**	COC1=C(OC)C=C(C=C1)C1CC(=NN1)C1=C2C=CC=CC2=CC2=C1C=CC=C2	0,25	μM	IC50	HeLa
**255.**	COC1=CC=C(C=C1)C1CC(=NN1)C1=C2C=CC=CC2=CC2=C1C=CC=C2	0,25	μM	IC50	HeLa
**256.**	C1C(NN=C1C1=C2C=CC=CC2=CC2=C1C=CC=C2)C1=CC=NC=C1	35	μM	IC50	HeLa
**257.**	COC1=CC(OC)=C(C=C1)C(=O)\C=C\C1=CC(OC)=C(OC)C(OC)=C1	3,2	μM	IC50	HeLa
**258.**	COC1=C(O)C(C(=O)\C=C\C2=CC=C(O)C=C2)=C(OC)C(OC)=C1OC	34,09	μM	IC50	HeLa
**259.**	COC1=CC(\C=C\C(=O)C2=C(OC)C(OC)=C(OC)C(OC)=C2O)=CC=C1O	29,23	μM	IC50	HeLa
**260.**	COC1=CC=C(\C=C\C(=O)C2=C(OC)C(OC)=C(OC)C(OC)=C2O)C=C1O	1,44	μM	IC50	HeLa
**261.**	COC1=C(O)C(O)=CC(\C=C\C(=O)C2=C(OC)C(OC)=C(OC)C(OC)=C2O)=C1	32,95	μM	IC50	HeLa
**262.**	COC1=CC(\C=C\C(=O)C2=C(OC)C(OC)=C(OC)C(OC)=C2O)=CC(OC)=C1OC	4,99	μM	IC50	HeLa
**263.**	COC1=CC=C(C=C1)C1ON=C2C1SC(N2C1=CC=C(Cl)C=C1)C1=CC(CC2=CC(C3SC4C(ON=C4N3C3=CC=C(Cl)C=C3)C3=CC=C(OC)C=C3)=C(OC)C=C2)=CC=C1OC	>100	μg/mL	IC50	HeLa
**264.**	[H]C1CC[C@]2([H])[C@]1([H])CC[C@]1([H])C3=C(CC[C@@]21[H])C=C1OC(=CC(=O)C1=C3)C1=CC=CC=C1	3,9	μM	IC50	HeLa
**265.**	[H]C1CC[C@]2([H])[C@]1([H])CC[C@]1([H])C3=C(CC[C@@]21[H])C=C1OC(=CC(=O)C1=C3)C1=CC=C(C)C=C1	6,3	μM	IC50	HeLa
**266.**	[H]C1CC[C@]2([H])[C@]1([H])CC[C@]1([H])C3=C(CC[C@@]21[H])C=C1OC(=CC(=O)C1=C3)C1=CC=C(Br)C=C1	6,9	μM	IC50	HeLa

**267.**	COC1=CC=C(\C=C\C(=O)C2=CC(OC)=C(OC)C(OC)=C2)C=C1OC(=O)CCC(=O)O[Pt]([NH3])([NH3])(O)(Cl)Cl	0,41	µM	IC50	HeLa
**268.**	COC1=CC=C(\C=C\C(=O)C2=CC(OC)=C(OC)C(OC)=C2)C=C1OC(=O)CCC(=O)O[Pt]([NH3])([NH3])(Cl)(Cl)Cl	0,5	µM	IC50	HeLa
**269.**	COC1=CC=C(\C=C\C(=O)C2=CC(OC)=C(OC)C(OC)=C2)C=C1OC(=O)CCCC(=O)O[Pt]([NH3])([NH3])(O)(Cl)Cl	0,32	µM	IC50	HeLa
**270.**	COC1=CC=C(\C=C\C(=O)C2=CC(OC)=C(OC)C(OC)=C2)C=C1OC(=O)CCCC(=O)O[Pt]([NH3])([NH3])(Cl)(Cl)Cl	0,43	µM	IC50	HeLa
**271.**	COC1=CC(\C=C\C(=O)C2=CC=C(NC(=O)C3=CC=CC=C3CN3C(=O)C4=C(C=CC=C4)C3=O)C=C2)=CC(OC)=C1OC	1,62	µM	IC50	Hep G2
**272.**	O=C(NC1=CC=C(C=C1)C(=O)\C=C\C1=CC=CC=C1)C1=CC=CC=C1CC1C(=O)C2=C(C=CC=C2)C1=O	12,93	µM	IC50	Hep G2
**273.**	ClC1=CC=C(\C=C\C(=O)C2=CC=C(NC(=O)C3=CC=CC=C3CC3C(=O)C4=C(C=CC=C4)C3=O)C=C2)C=C1	3,21	µM	IC50	Hep G2
**274.**	COC1=CC=C(\C=C\C(=O)C2=CC=C(NC(=O)C3=CC=CC=C3CC3C(=O)C4=C(C=CC=C4)C3=O)C=C2)C=C1	1,87	µM	IC50	Hep G2
**275.**	O=C(NC1=CC=C(C=C1)C(=O)\C=C\C1=CC=C2C=CC=CC2=C1)C1=CC=CC=C1CC1C(=O)C2=C(C=CC=C2)C1=O	8,07	µM	IC50	Hep G2
**276.**	[O-][N+](=O)C1=CC=C(\C=C\C(=O)C2=CC=C(NC(=O)C3=CC=CC=C3CC3C(=O)C4=C(C=CC=C4)C3=O)C=C2)C=C1	8,47	µM	IC50	Hep G2
**277.**	COC1=CC=C(\C=C\C(=O)C2=CC=C(NC(=O)C3=CC=CC=C3CC3C(=O)C4=C(C=CC=C4)C3=O)C=C2)C(OC)=C1	15,85	µM	IC50	Hep G2
**278.**	O=C(NC1=CC=C(C=C1)C(=O)\C=C\C1=CC=CO1)C1=CC=CC=C1CC1C(=O)C2=C(C=CC=C2)C1=O	1,75	µM	IC50	Hep G2
**279.**	C1C(NN=C1C1=C2C=CC=CC2=CC2=C1C=CC=C2)C1=CC=CC=C1	3,86	µM	IC50	HepG2
**280.**	ClC1=CC=C(C=C1)C1CC(=NN1)C1=C2C=CC=CC2=CC2=C1C=CC=C2	22,22	µM	IC50	HepG2
**281.**	FC1=CC=C(C=C1)C1CC(=NN1)C1=C2C=CC=CC2=CC2=C1C=CC=C2	3,5	µM	IC50	HepG2

**282.**	CC1=CC=C(C=C1)C1CC(=NN1)C1=C2C=CC=CC2=CC2=C1C=CC=C2	26,44	µM	IC50	HepG2
**283.**	ClC1=CC(=CC=C1)C1CC(=NN1)C1=C2C=CC=CC2=CC2=C1C=CC=C2	20,88	µM	IC50	HepG2
**284.**	[O-<br>][N+](=O)C1=CC=C(C=C1)C1CC(=NN1)C1=C2C=CC=CC2=CC2=C1C=CC=C2	20,65	µM	IC50	HepG2
**285.**	BrC1=CC=C(C=C1)C1CC(=NN1)C1=C2C=CC=CC2=CC2=C1C=CC=C2	19,22	µM	IC50	HepG2
**286.**	FC1=CC(=CC=C1)C1CC(=NN1)C1=C2C=CC=CC2=CC2=C1C=CC=C2	33,44	µM	IC50	HepG2
**287.**	[O-<br>][N+](=O)C1=CC(=CC=C1)C1CC(=NN1)C1=C2C=CC=CC2=CC2=C1C=CC=C2	0,22	µM	IC50	HepG2
**288.**	CN(C)C1=CC=C(C=C1)C1CC(=NN1)C1=C2C=CC=CC2=CC2=C1C=CC=C2	1,58	µM	IC50	HepG2
**289.**	C1C(NN=C1C1=C2C=CC=CC2=CC2=C1C=CC=C2)C1=CC=C(C=C1)C1=CC=C<br>O1	0,44	µM	IC50	HepG2
**290.**	C1C(NN=C1C1=C2C=CC=CC2=CC2=C1C=CC=C2)C1=CC=CO1	0,4	µM	IC50	HepG2
**291.**	COC1=CC(=CC(OC)=C1OC)C1CC(=NN1)C1=C2C=CC=CC2=CC2=C1C=CC=<br>C2	0,22	µM	IC50	HepG2
**292.**	COC1=C(OC)C=C(C=C1)C1CC(=NN1)C1=C2C=CC=CC2=CC2=C1C=CC=C2	2,66	µM	IC50	HepG2
**293.**	COC1=CC=C(C=C1)C1CC(=NN1)C1=C2C=CC=CC2=CC2=C1C=CC=C2	3,26	µM	IC50	HepG2
**294.**	C1C(NN=C1C1=C2C=CC=CC2=CC2=C1C=CC=C2)C1=CC=NC=C1	15,23	µM	IC50	HepG2
**295.**	O=C(C1CC2CSCN2C11C(=O)NC2=C1C=CC=C2)C1=CNC2=C1C=CC=C2	5.5 ± 0.2	µM	IC50	HepG2
**296.**	[H]C1=C(OC)C([H])=C2C=CC(=[O+]C2=C1[H])C1=CC=C(O)C(OC)=C1	70	%<br>viabilidade	%	HepG2
**297.**	COC1=CC(OC2=CC(OC)=C(OC)C(OC)=C2)=C(C(/C=C/C3=CC=C(OC)C=C3)=<br>O)C=C1	4,64	µM	IC50	HepG2
**298.**	CCOC(=O)CN1C(=O)C2=C(SC3=C2CCN(C3)C(=O)OCC)N=C1SCC(=O)NC1=<br>CC=C(C=C1)C(=O)\C=C/C1=CC=CC=C1Cl	18,5	%	% sobrevivência	HepG2
**299.**	CCOC(=O)CN1C(=O)C2=C(SC3=C2CCN(C3)C(=O)OCC)N=C1SCC(=O)NC1=<br>CC=C(C=C1)C(=O)\C=C/C1=CC(OC)=C(OC)C(OC)=C1	12,1	%	% sobrevivência	HepG2
**300.**	CCOC(=O)CN1C(=O)C2=C(SC3=C2CCN(C3)C(=O)OCC)N=C1SCC(=O)NC1=<br>CC=C(C=C1)C(=O)\C=C/C1=CC=CC(=C1)[N+]([O-])=O	18	%	% sobrevivência	HepG2

**301.**	CCOCC1=NC2=C(C3=C(CN(CC3)C(=O)OCC)S2)C(=O)N1CC(=O)NC1=CC=C(C=C1)C(=O)\C=C/C1=CC=C(OC)C(OC)=C1OC	18,4	%	% sobrevivência	HepG2
**302.**	CCOC(=O)N1CCC2=C(C1)SC1=C2C(=O)NC(SCC(=O)NC2=CC=C(C=C2)C(=O)\C=C/C2=CC(OC)=C(OC)C(OC)=C2)=N1	14,1	%	% sobrevivência	HepG2
**303.**	CCOCC1=NC2=C(C3=C(CN(CC3)C(=O)OCC)S2)C(=O)N1CC(=O)NC1=CC=C(C=C1)C(=O)\C=C/C1=CC=CC(=C1)[N+]([O-])=O	10,7	%	% sobrevivência	HepG2
**304.**	CCOC(=O)CN1C(=O)C2=C(SC3=C2CC(C)(C)NC3(C)C)N=C1SCC(=O)NC1=CC=C(C=C1)C(=O)\C=C/C1=CC=CC(=C1)[N+]([O-])=O	12,3	%	% sobrevivência	HepG2
**305.**	CC1=CC=C(C=C1)C(=O)\C=C\C1=CC=C(\C=N\NC(N)=N)C=C1	7,17	μM	IC50	HepG2
**306.**	COC1=CC=C(\C=C\C(=O)C2=CC(OC)=C(OC)C(OC)=C2)C=C1OC(=O)CCC(=O)O[Pt]([NH3])([NH3])(O)(Cl)Cl	0,33	μM	IC50	HepG-2
**307.**	COC1=CC=C(\C=C\C(=O)C2=CC(OC)=C(OC)C(OC)=C2)C=C1OC(=O)CCC(=O)O[Pt]([NH3])([NH3])(Cl)(Cl)Cl	0,41	μM	IC50	HepG-2
**308.**	COC1=CC=C(\C=C\C(=O)C2=CC(OC)=C(OC)C(OC)=C2)C=C1OC(=O)CCCC(=O)O[Pt]([NH3])([NH3])(O)(Cl)Cl	0,29	μM	IC50	HepG-2
**309.**	COC1=CC=C(\C=C\C(=O)C2=CC(OC)=C(OC)C(OC)=C2)C=C1OC(=O)CCCC(=O)O[Pt]([NH3])([NH3])(Cl)(Cl)Cl	0,35	μM	IC50	HepG-2
**310.**	[H]OC1=C(C=CC=C1)C(=O)\C=C\C1=CC=C(OC)C=C1	35,2	μM	IC50	HL60
**311.**	COC1=CC=C(\C=C\C(=O)C2=C(OC(=O)C3=CC=CO3)C=CC=C2)C=C1	0,3	μM	IC50	HL60
**312.**	[H]N([H])C1=C(C=CC=C1)C(=O)\C=C\C1=CC=C(OC)C=C1	12,4	μM	IC50	HL60
**313.**	[H]N(C(=O)C1=CC=CO1)C1=C(C=CC=C1)C(=O)\C=C\C1=CC=C(OC)C=C1	0,5	μM	IC50	HL60
**314.**	[H]OC1=C(C=CC=C1)C(=O)\C=C\C1=CC(OC)=C(OC)C(OC)=C1	46,6	μM	IC50	HL60
**315.**	COC1=CC(\C=C\C(=O)C2=C(OC(=O)C3=CC=CO3)C=CC=C2)=CC(OC)=C1OC	23,1	μM	IC50	HL60
**316.**	[H]OC1=C(C(=O)\C=C\C2=CC(OC)=C(OC)C(OC)=C2)C(OBr)=CC=C1	5,8	μM	IC50	HL60
**317.**	COC1=CC(\C=C\C(=O)C2=C(OC(=O)C3=CC=CO3)C=CC=C2OBr)=CC(OC)=C1OC	3,6	μM	IC50	HL60
**318.**	[H]N([H])C1=C(C=CC=C1)C(=O)\C=C\C1=CC(OC)=C(OC)C(OC)=C1	6,3	μM	IC50	HL60

**319.**	[H]N(C(=O)C1=CC=CO1)C1=C(C=CC=C1)C(=O)\C=C\C1=CC(OC)=C(OC)C(OC)=C1	26,1	μM	IC50	HL60
**320.**	[H]OC1=C(C=CC=C1)C(=O)\C=C\C1=CC(OC)=C(OC)C(OC)=C1	>100	μM	IC50	HL60
**321.**	COC1=CC(\C=C\C(=O)C2=C(OC(=O)C3=CC=CO3)C=CC=C2)=CC(OC)=C1OC	7,8	μM	IC50	HL60
**322.**	[H]OC1=C(OC2=C(C=CC=C2)C1=O)C1=CC=C(OC)C=C1	55,9	μM	IC50	HL60
**323.**	COC1=CC=C(C=C1)C1=C(OC(=O)C2=CC=CO2)C(=O)C2=C(O1)C=CC=C2	33,1	μM	IC50	HL60
**324.**	COC1=CC(\C=C\C(=O)C2=CC=C3OC(=O)N(CN4CCCCC4)C3=C2)=CC(OC)=C1OC	5,4	μM	IC50	HL-60
**325.**	COC1=CC(\C=C\C(=O)C2=CC=C3OC(=O)N(CN4CCOCC4)C3=C2)=CC(OC)=C1OC	8,9	μM	IC50	HL-60
**326.**	COC1=CC(\C=C\C(=O)C2=CC=C3OC(=O)N(CN4CCSCC4)C3=C2)=CC(OC)=C1OC	5,6	μM	IC50	HL-60
**327.**	COC1=CC(\C=C\C(=O)C2=CC=C3OC(=O)N(CN4CCN(CC5=CC=CC=C5)CC4)C3=C2)=CC(OC)=C1OC	7,1	μM	IC50	HL-60
**328.**	COC1=CC(\C=C\C(=O)C2=CC=C3OC(=O)N(CN4CCN(CC4)C4=C(OC)C=CC=C4)C3=C2)=CC(OC)=C1OC	20,7	μM	IC50	HL-60
**329.**	[H]C1=C([H])C(=CC=C1\C=C\C(=C)C1=C(C)N(N=N1)C1=CC=C(Br)C=C1)N(C)C	77,05	μM	IC50	HL-60 (TB)
**330.**	[H]C1=C(OC)C(OC)=CC=C1\C=C\C(=C)C1=C(C)N(N=N1)C1=CC=C(Br)C=C1	47,12	μM	IC50	HL-60 (TB)
**331.**	[H]C1=C([H])C(\C=C\C(=O)C2=C(C)N(N=N2)C2=CC=C(Br)C=C2)=CC=C1OC	94,21	μM	IC50	HL-60 (TB)
**332.**	[H]C1=CC=C(\C=C\C(=O)C2=C(C)N(N=N2)C2=CC=C(Br)C=C2)C(OC)=C1[H]	59,81	μM	IC50	HL-60 (TB)
**333.**	[H]C1=CC=C(\C=C\C(=O)C2=C(C)N(N=N2)C2=CC=C(Br)C=C2)C([H])=C1[N+]([O-])=O	76,2	μM	IC50	HL-60 (TB)

**334.**	[H]C1=C([H])C(\C=C\C(=O)C2=C(C)N(N=N2)C2=CC=C(Br)C=C2)=CC=C1Cl	105,34	µM	IC50	HL-60 (TB)
**335.**	[H]C1=C([H])C(=CC=C1\C=C\C(=O)C1=C(C)N(N=N1)C1=CC=C(F)C=C1)N(C)C	103,92	µM	IC50	HL-60 (TB)
**336.**	[H]C1=C(OC)C(OC)=CC=C1\C=C\C(=O)C1=C(C)N(N=N1)C1=CC=C(F)C=C1	58,46	µM	IC50	HL-60 (TB)
**337.**	[H]C1=C([H])C(\C=C\C(=O)C2=C(C)N(N=N2)C2=CC=C(F)C=C2)=CC=C1OC	92,94	µM	IC50	HL-60 (TB)
**338.**	[H]C1=CC=C(\C=C\C(=O)C2=C(C)N(N=N2)C2=CC=C(F)C=C2)C(OC)=C1[H]	31,92	µM	IC50	HL-60 (TB)
**339.**	[H]C1=C([H])C(=CC=C1\C=C\C(=O)C1=C(C)N(N=N1)C1=CC=C(C=C1)C(O)=O)N(C)C	82,93	µM	IC50	HL-60 (TB)
**340.**	[H]C1=C(OC)C(OC)=CC=C1\C=C\C(=O)C1=C(C)N(N=N1)C1=CC=C(C=C1)C(O)=O	83,19	µM	IC50	HL-60 (TB)
**341.**	[H]C1=C([H])C(\C=C\C(=O)C2=C(C)N(N=N2)C2=CC=C(C=C2)C(O)=O)=CC=C1OC	101,94	µM	IC50	HL-60 (TB)
**342.**	[H]C1=CC=C(\C=C\C(=O)C2=C(C)N(N=N2)C2=CC=C(C=C2)C(O)=O)C(OC)=C1[H]	83,2	µM	IC50	HL-60 (TB)
**343.**	COC1=CC=C(\C=C\C(=O)C2=CC(OC)=C(OC)C(OC)=C2)C=C1OC(=O)CCC(=O)O[Pt]([NH3])([NH3])(O)(Cl)Cl	11,85	µM	IC50	HL-7702
**344.**	COC1=CC=C(\C=C\C(=O)C2=CC(OC)=C(OC)C(OC)=C2)C=C1OC(=O)CCC(=O)O[Pt]([NH3])([NH3])(Cl)(Cl)Cl	12,3	µM	IC50	HL-7702
**345.**	COC1=CC=C(\C=C\C(=O)C2=CC(OC)=C(OC)C(OC)=C2)C=C1OC(=O)CCCC(=O)O[Pt]([NH3])([NH3])(O)(Cl)Cl	12,35	µM	IC50	HL-7702
**346.**	COC1=CC=C(\C=C\C(=O)C2=CC(OC)=C(OC)C(OC)=C2)C=C1OC(=O)CCCC(=O)O[Pt]([NH3])([NH3])(Cl)(Cl)Cl	13,08	µM	IC50	HL-7702
**347.**	[H]C1=C([H])C(=CC=C1\C=C\C(=C)C1=C(C)N(N=N1)C1=CC=C(Br)C=C1)N(C)C	103,69	µM	IC50	HOP-62

**348.**	[H]C1=C(OC)C(OC)=CC=C1\C=C\C(=C)C1=C(C)N(N=N1)C1=CC=C(Br)C=C1	88,14	μM	IC50	HOP-62
**349.**	[H]C1=C([H])C(\C=C\C(=O)C2=C(C)N(N=N2)C2=CC=C(Br)C=C2)=CC=C1OC	95,48	μM	IC50	HOP-62
**350.**	[H]C1=CC=C(\C=C\C(=O)C2=C(C)N(N=N2)C2=CC=C(Br)C=C2)C(OC)=C1[H]	113,22	μM	IC50	HOP-62
**351.**	[H]C1=CC=C(\C=C\C(=O)C2=C(C)N(N=N2)C2=CC=C(Br)C=C2)C([H])=C1[N+]([O-])=O	101,35	μM	IC50	HOP-62
**352.**	[H]C1=C([H])C(\C=C\C(=O)C2=C(C)N(N=N2)C2=CC=C(Br)C=C2)=CC=C1Cl	100,29	μM	IC50	HOP-62
**353.**	[H]C1=C([H])C(=CC=C1\C=C\C(=O)C1=C(C)N(N=N1)C1=CC=C(F)C=C1)N(C)C	102,98	μM	IC50	HOP-62
**354.**	[H]C1=C(OC)C(OC)=CC=C1\C=C\C(=O)C1=C(C)N(N=N1)C1=CC=C(F)C=C1	103,82	μM	IC50	HOP-62
**355.**	[H]C1=C([H])C(\C=C\C(=O)C2=C(C)N(N=N2)C2=CC=C(F)C=C2)=CC=C1OC	92,77	μM	IC50	HOP-62
**356.**	[H]C1=CC=C(\C=C\C(=O)C2=C(C)N(N=N2)C2=CC=C(F)C=C2)C(OC)=C1[H]	72,16	μM	IC50	HOP-62
**357.**	[H]C1=C([H])C(=CC=C1\C=C\C(=O)C1=C(C)N(N=N1)C1=CC=C(C=C1)C(O)=O)N(C)C	93,41	μM	IC50	HOP-62
**358.**	[H]C1=C(OC)C(OC)=CC=C1\C=C\C(=O)C1=C(C)N(N=N1)C1=CC=C(C=C1)C(O)=O	107,42	μM	IC50	HOP-62
**359.**	[H]C1=C([H])C(\C=C\C(=O)C2=C(C)N(N=N2)C2=CC=C(C=C2)C(O)=O)=CC=C1OC	101,53	μM	IC50	HOP-62
**360.**	[H]C1=CC=C(\C=C\C(=O)C2=C(C)N(N=N2)C2=CC=C(C=C2)C(O)=O)C(OC)=C1[H]	110,05	μM	IC50	HOP-62
**361.**	[H]C1=C([H])C(=CC=C1\C=C\C(=C)C1=C(C)N(N=N1)C1=CC=C(Br)C=C1)N(C)C	81,67	μM	IC50	HOP-92

**362.**	[H]C1=C(OC)C(OC)=CC=C1\C=C\C(=C)C1=C(C)N(N=N1)C1=CC=C(Br)C=C1	81	μM	IC50	HOP-92
**363.**	[H]C1=C([H])C(\C=C\C(=O)C2=C(C)N(N=N2)C2=CC=C(Br)C=C2)=CC=C1OC	86,95	μM	IC50	HOP-92
**364.**	[H]C1=CC=C(\C=C\C(=O)C2=C(C)N(N=N2)C2=CC=C(Br)C=C2)C(OC)=C1[H]	84,56	μM	IC50	HOP-92
**365.**	[H]C1=CC=C(\C=C\C(=O)C2=C(C)N(N=N2)C2=CC=C(Br)C=C2)C([H])=C1[N+]([O-])=O	95,61	μM	IC50	HOP-92
**366.**	[H]C1=C([H])C(\C=C\C(=O)C2=C(C)N(N=N2)C2=CC=C(Br)C=C2)=CC=C1Cl	83,96	μM	IC50	HOP-92
**367.**	[H]C1=C([H])C(=CC=C1\C=C\C(=O)C1=C(C)N(N=N1)C1=CC=C(F)C=C1)N(C)C	92,33	μM	IC50	HOP-92
**368.**	[H]C1=C(OC)C(OC)=CC=C1\C=C\C(=O)C1=C(C)N(N=N1)C1=CC=C(F)C=C1	98,54	μM	IC50	HOP-92
**369.**	[H]C1=C([H])C(\C=C\C(=O)C2=C(C)N(N=N2)C2=CC=C(F)C=C2)=CC=C1OC	72,9	μM	IC50	HOP-92
**370.**	[H]C1=CC=C(\C=C\C(=O)C2=C(C)N(N=N2)C2=CC=C(F)C=C2)C(OC)=C1[H]	91,08	μM	IC50	HOP-92
**371.**	[H]C1=C([H])C(=CC=C1\C=C\C(=O)C1=C(C)N(N=N1)C1=CC=C(C=C1)C(O)=O)N(C)C	101,5	μM	IC50	HOP-92
**372.**	[H]C1=C(OC)C(OC)=CC=C1\C=C\C(=O)C1=C(C)N(N=N1)C1=CC=C(C=C1)C(O)=O	94,7	μM	IC50	HOP-92
**373.**	[H]C1=C([H])C(\C=C\C(=O)C2=C(C)N(N=N2)C2=CC=C(C=C2)C(O)=O)=CC=C1OC	101,29	μM	IC50	HOP-92
**374.**	[H]C1=CC=C(\C=C\C(=O)C2=C(C)N(N=N2)C2=CC=C(C=C2)C(O)=O)C(OC)=C1[H]	92,33	μM	IC50	HOP-92
**375.**	COC1=CC(OC)=C(C=C1S(N)(=O)=O)C(=O)\C=C\C1=CC=C(Cl)C=C1	1,46	μM	IC50	HOP-92
**376.**	COC1=CC(OC)=C(C=C1)C(=O)\C=C\C1=CC(OC)=C(OC)C(OC)=C1	10,8	μM	IC50	HT-1376

**377.**	CC(C)CC1=CC=C(C=C1)C1=NC2=C(SC(C1)C1=CC=CN1)C=CC=C2	16	µg/mL	IC50	HT-29
**378.**	CC(C)CC1=CC=C(C=C1)C1=NC2=C(SC(C1)C1=CC=CS1)C=CC=C2	18	µg/mL	IC50	HT-29
**379.**	CC(C)CC1=CC=C(C=C1)C1=NC2=C(SC(C1)C1=CC=C(Br)O1)C=CC=C2	2	µg/mL	IC50	HT-29
**380.**	[H]C1=CC(\C=C\C(=O)C2=CC=C(C=C2)N2C=C(CN3CCN(CC3)C3=CC4=C(C=C3F)C(=O)C(=CN4C3CC3)C(O)=O)N=N2)=CC([H])=C1[H]	22,35	µM	IC50	HT-29
**381.**	[H]C1=C([H])C(Cl)=CC(\C=C\C(=O)C2=CC=C(C=C2)N2C=C(CN3CCN(CC3)C3=CC4=C(C=C3F)C(=O)C(=CN4C3CC3)C(O)=O)N=N2)=C1	42,91	µM	IC50	HT-29
**382.**	[H]C1=CC(\C=C\C(=O)C2=CC=C(C=C2)N2C=C(CN3CCN(CC3)C3=CC4=C(C=C3F)C(=O)C(=CN4C3CC3)C(O)=O)N=N2)=CC([H])=C1F	8,67	µM	IC50	HT-29
**383.**	[H]C1=C(OC)C(OC)=CC(\C=C\C(=O)C2=CC=C(C=C2)N2C=C(CN3CCN(CC3)C3=CC4=C(C=C3F)C(=O)C(=CN4C3CC3)C(O)=O)N=N2)=C1	18,6	µM	IC50	HT-29
**384.**	COC1=CC(\C=C\C(=O)C2=CC=C(C=C2)N2C=C(CN3CCN(CC3)C3=CC4=C(C=C3F)C(=O)C(=CN4C3CC3)C(O)=O)N=N2)=CC(OC)=C1OC	13,24	µM	IC50	HT-29
**385.**	CC1=NC(C)=C(C)N=C1\C=C\C(=O)C1=CC=CO1	9,58	µM	IC50	HT-29
**386.**	COC1=CC=C2OC(=CC2=C1)C(=O)\C=C\C1=C(C)N=C(C)C(C)=N1	8,18	µM	IC50	HT-29
**387.**	CC1=CSC(=C1)C1=CC=C(C=C1)C(=O)\C=C\C1=NC(C)=C(C)N=C1C	6,54	µM	IC50	HT-29
**388.**	CN(C)C1=CC=C(\C=C\C(=O)C2=C(C)NC(=O)NC2C2=C3OC(=O)C=C(O)C3=CC=C2)C=C1	65,56	µg/mL	IC50	jurkat
**389.**	CON1C=C(C(=O)\C=C\C2=C(OC)C=C(OC)C=C2OC)C2=CC=CC=C12	< 7	µM	IC50	Jurkat
**390.**	COC1=CC=C(\C=C\C(=O)C2=C(C)N(N=N2)C2=CC=C(Cl)C=C2)C=C1OC	0,84	µM	IC50	K-562
**391.**	[H]C1=C(OC)C(OC)=CC=C1\C=C\C(=C)C1=C(C)N(N=N1)C1=CC=C(Br)C=C1	82,02	µM	IC50	K-562
**392.**	[H]C1=C([H])C(\C=C\C(=O)C2=C(C)N(N=N2)C2=CC=C(Br)C=C2)=CC=C1OC	19,15	µM	IC50	K-562
**393.**	[H]C1=CC=C(\C=C\C(=O)C2=C(C)N(N=N2)C2=CC=C(Br)C=C2)C(OC)=C1[H]	90,22	µM	IC50	K-562
**394.**	[H]C1=CC=C(\C=C\C(=O)C2=C(C)N(N=N2)C2=CC=C(Br)C=C2)C([H])=C1[N+]([O-])=O	74,8	µM	IC50	K-562

**395.**	[H]C1=C([H])C(\C=C\C(=O)C2=C(C)N(N=N2)C2=CC=C(Br)C=C2)=CC=C1Cl	75,55	μM	IC50	K-562
**396.**	[H]C1=C([H])C(=CC=C1\C=C\C(=O)C1=C(C)N(N=N1)C1=CC=C(F)C=C1)N(C)C	96,59	μM	IC50	K-562
**397.**	[H]C1=C(OC)C(OC)=CC=C1\C=C\C(=O)C1=C(C)N(N=N1)C1=CC=C(F)C=C1	101,69	μM	IC50	K-562
**398.**	[H]C1=C([H])C(\C=C\C(=O)C2=C(C)N(N=N2)C2=CC=C(F)C=C2)=CC=C1OC	50,65	μM	IC50	K-562
**399.**	[H]C1=CC=C(\C=C\C(=O)C2=C(C)N(N=N2)C2=CC=C(F)C=C2)C(OC)=C1[H]	97,28	μM	IC50	K-562
**400.**	[H]C1=C([H])C(=CC=C1\C=C\C(=O)C1=C(C)N(N=N1)C1=CC=C(C=C1)C(O)=O)N(C)C	8,81	μM	IC50	K-562
**401.**	[H]C1=C(OC)C(OC)=CC=C1\C=C\C(=O)C1=C(C)N(N=N1)C1=CC=C(C=C1)C(O)=O	98,48	μM	IC50	K-562
**402.**	[H]C1=C([H])C(\C=C\C(=O)C2=C(C)N(N=N2)C2=CC=C(C=C2)C(O)=O)=CC=C1OC	80,83	μM	IC50	K-562
**403.**	[H]C1=CC=C(\C=C\C(=O)C2=C(C)N(N=N2)C2=CC=C(C=C2)C(O)=O)C(OC)=C1[H]	88,58	μM	IC50	K-562
**404.**	FC(F)(F)C1=CC=C(C=C1)C1C2CSCN2C2(C1C(=O)C1=CNC3=C1C=CC=C3)C(=O)NC1=C2C=CC=C1	0,622	μM	GI50	K-562
**405.**	COC1=CC(\C=C\C(=O)C2=CC=C3OC(=O)N(CN4CCCCC4)C3=C2)=CC(OC)=C1OC	10,7	μM	IC50	K-562
**406.**	COC1=CC(\C=C\C(=O)C2=CC=C3OC(=O)N(CN4CCOCC4)C3=C2)=CC(OC)=C1OC	9,7	μM	IC50	K-562
**407.**	COC1=CC(\C=C\C(=O)C2=CC=C3OC(=O)N(CN4CCSCC4)C3=C2)=CC(OC)=C1OC	7,1	μM	IC50	K-562
**408.**	COC1=CC(\C=C\C(=O)C2=CC=C3OC(=O)N(CN4CCN(CC5=CC=CC=C5)CC4)C3=C2)=CC(OC)=C1OC	10,7	μM	IC50	K-562

**409.**	COC1=CC(\C=C\C(=O)C2=CC=C3OC(=O)N(CN4CCN(CC4)C4=C(OC)C=C C=C4)C3=C2)=CC(OC)=C1OC	8,9	μM	IC50	K-562
**410.**	COC1=CC(OC)=C(C=C1S(N)(=O)=O)C(=O)\C=C\C1=CC=CC=C1	1,5	μM	IC50	K-562
**411.**	CCCCN1C(=O)C(\C=C\C(=O)\C=C\C2=CC3=C(C=CC=C3)N(CCCC)C2=O) =CC2=C1C=CC=C2	0,88	μM	GI50	K-562
**412.**	COC1=CC=C(\C=C\C(=O)C2=C(C)N(N=N2)C2=CC=C(Cl)C=C2)C=C1OC	0,96	μM	IC50	Leucemia - SR
**413.**	[H]C1=C([H])C(=CC=C1\C=C\C(=C)C1=C(C)N(N=N1)C1=CC=C(Br)C=C1)N (C)C	95,35	μM	IC50	Leucemia - SR
**414.**	[H]C1=C(OC)C(OC)=CC=C1\C=C\C(=C)C1=C(C)N(N=N1)C1=CC=C(Br)C=C 1	66,41	μM	IC50	Leucemia - SR
**415.**	[H]C1=C([H])C(\C=C\C(=O)C2=C(C)N(N=N2)C2=CC=C(Br)C=C2)=CC=C1O C	99,13	μM	IC50	Leucemia - SR
**416.**	[H]C1=CC=C(\C=C\C(=O)C2=C(C)N(N=N2)C2=CC=C(Br)C=C2)C(OC)=C1[H]	83,82	μM	IC50	Leucemia - SR
**417.**	[H]C1=CC=C(\C=C\C(=O)C2=C(C)N(N=N2)C2=CC=C(Br)C=C2)C([H])=C1[N+]([O-])=O	88,77	μM	IC50	Leucemia - SR
**418.**	[H]C1=C([H])C(\C=C\C(=O)C2=C(C)N(N=N2)C2=CC=C(Br)C=C2)=CC=C1C l	98,56	μM	IC50	Leucemia - SR
**419.**	[H]C1=C([H])C(=CC=C1\C=C\C(=O)C1=C(C)N(N=N1)C1=CC=C(F)C=C1)N(C)C	95,77	μM	IC50	Leucemia - SR
**420.**	[H]C1=C(OC)C(OC)=CC=C1\C=C\C(=O)C1=C(C)N(N=N1)C1=CC=C(F)C=C 1	79,61	μM	IC50	Leucemia - SR
**421.**	[H]C1=C([H])C(\C=C\C(=O)C2=C(C)N(N=N2)C2=CC=C(F)C=C2)=CC=C1O C	99,2	μM	IC50	Leucemia - SR
**422.**	[H]C1=CC=C(\C=C\C(=O)C2=C(C)N(N=N2)C2=CC=C(F)C=C2)C(OC)=C1[H]	14,01	μM	IC50	Leucemia - SR

**423.**	[H]C1=C([H])C(=CC=C1\C=C\C(=O)C1=C(C)N(N=N1)C1=CC=C(C=C1)C(O)=O)N(C)C	97,44	µM	IC50	Leucemia - SR
**424.**	[H]C1=C(OC)C(OC)=CC=C1\C=C\C(=O)C1=C(C)N(N=N1)C1=CC=C(C=C1)C(O)=O	87,73	µM	IC50	Leucemia - SR
**425.**	[H]C1=C([H])C(\C=C\C(=O)C2=C(C)N(N=N2)C2=CC=C(C=C2)C(O)=O)=CC=C1OC	96,29	µM	IC50	Leucemia - SR
**426.**	[H]C1=CC=C(\C=C\C(=O)C2=C(C)N(N=N2)C2=CC=C(C=C2)C(O)=O)C(OC)=C1[H]	63,92	µM	IC50	Leucemia - SR
**427.**	COC1=CC(OC)=C(C=C1S(N)(=O)=O)C(=O)\C=C\C1=CC=C(C)C=C1	0,54	µM	IC50	LOX IMVI
**428.**	O=C(NC1=CC=C(C=C1)C(=O)\C=C\C1=CC=CC=C1)C1=CC=CC=C1CC1C(=O)C2=C(C=CC=C2)C1=O	14,43	µM	IC50	MCF -7
**429.**	ClC1=CC=C(\C=C\C(=O)C2=CC=C(NC(=O)C3=CC=CC=C3CC3C(=O)C4=C(C=CC=C4)C3=O)C=C2)C=C1	4,11	µM	IC50	MCF -7
**430.**	COC1=CC=C(\C=C\C(=O)C2=CC=C(NC(=O)C3=CC=CC=C3CC3C(=O)C4=C(C=CC=C4)C3=O)C=C2)C=C1	2,41	µM	IC50	MCF -7
**431.**	O=C(NC1=CC=C(C=C1)C(=O)\C=C\C1=CC=C2C=CC=CC2=C1)C1=CC=CC=C1CC1C(=O)C2=C(C=CC=C2)C1=O	11,31	µM	IC50	MCF -7
**432.**	[O-][N+](=O)C1=CC=C(\C=C\C(=O)C2=CC=C(NC(=O)C3=CC=CC=C3CC3C(=O)C4=C(C=CC=C4)C3=O)C=C2)C=C1	5,28	µM	IC50	MCF -7
**433.**	COC1=CC=C(\C=C\C(=O)C2=CC=C(NC(=O)C3=CC=CC=C3CC3C(=O)C4=C(C=CC=C4)C3=O)C=C2)C(OC)=C1	9,78	µM	IC50	MCF -7
**434.**	O=C(NC1=CC=C(C=C1)C(=O)\C=C\C1=CC=CO1)C1=CC=CC=C1CC1C(=O)C2=C(C=CC=C2)C1=O	2,09	µM	IC50	MCF -7
**435.**	COC1=CC=C(C=C1)C1=CC2=CC=CC=C2N=C1\C=C\C(=O)C1=CC(OC)=C(OC)C(OC)=C1	1,05	µM	IC50	MCF-7
**436.**	CC(C)CC1=CC=C(C=C1)C1=NC2=C(SC(C1)C1=CC=CN1)C=CC=C2	15	µg/mL	IC50	MCF-7
**437.**	CC(C)CC1=CC=C(C=C1)C1=NC2=C(SC(C1)C1=CC=CS1)C=CC=C2	13	µg/mL	IC50	MCF-7

**438.**	CC(C)CC1=CC=C(C=C1)C1=NC2=C(SC(C1)C1=CC=C(Br)O1)C=CC=C2	2	µg/mL	IC50	MCF-7
**439.**	COC1=CC(OC)=C(C=C1)C(=O)\C=C\C1=CC(OC)=C(OC)C(OC)=C1	21,1	µM	IC50	MCF-7
**440.**	ClC1=CC(Cl)=C(C=C1)C(=O)\C=C\C1=CC=CS1	7,4	µM	IC50	MCF-7
**441.**	COC1=CC=C(C=C1)C1ON=C2C1SC(N2C1=CC=C(Cl)C=C1)C1=CC(CC2=CC(C3SC4C(ON=C4N3C3=CC=C(Cl)C=C3)C3=CC=C(OC)C=C3)=C(OC)C=C2)=CC=C1OC	55,16	µg/mL	IC50	MCF-7
**442.**	COC1=CC(OC2=CC(OC)=C(OC)C(OC)=C2)=C(C(/C=C/C3=CC=C(OC)C=C3)=O)C=C1	3,44	µM	IC50	MCF-7
**443.**	CCOC(=O)CN1C(=O)C2=C(SC3=C2CCN(C3)C(=O)OCC)N=C1SCC(=O)NC1=CC=C(C=C1)C(=O)\C=C/C1=CC=CC=C1Cl	6	%	% sobrevivência	MCF-7
**444.**	CCOC(=O)CN1C(=O)C2=C(SC3=C2CCN(C3)C(=O)OCC)N=C1SCC(=O)NC1=CC=C(C=C1)C(=O)\C=C/C1=CC(OC)=C(OC)C(OC)=C1	17,3	%	% sobrevivência	MCF-7
**445.**	CCOC(=O)CN1C(=O)C2=C(SC3=C2CCN(C3)C(=O)OCC)N=C1SCC(=O)NC1=CC=C(C=C1)C(=O)\C=C/C1=CC=CC(=C1)[N+]([O-])=O	5,9	%	% sobrevivência	MCF-7
**446.**	CCOCC1=NC2=C(C3=C(CN(CC3)C(=O)OCC)S2)C(=O)N1CC(=O)NC1=CC=C(C=C1)C(=O)\C=C/C1=CC=C(OC)C(OC)=C1OC	5,8	%	% sobrevivência	MCF-7
**447.**	CCOC(=O)N1CCC2=C(C1)SC1=C2C(=O)NC(SCC(=O)NC2=CC=C(C=C2)C(=O)\C=C/C2=CC(OC)=C(OC)C(OC)=C2)=N1	15,9	%	% sobrevivência	MCF-7
**448.**	CCOCC1=NC2=C(C3=C(CN(CC3)C(=O)OCC)S2)C(=O)N1CC(=O)NC1=CC=C(C=C1)C(=O)\C=C/C1=CC=CC(=C1)[N+]([O-])=O	4,6	%	% sobrevivência	MCF-7
**449.**	CCOC(=O)CN1C(=O)C2=C(SC3=C2CC(C)(C)NC3(C)C)N=C1SCC(=O)NC1=CC=C(C=C1)C(=O)\C=C/C1=CC=CC(=C1)[N+]([O-])=O	15,3	%	% sobrevivência	MCF-7
**450.**	COC1=CC(O)=C(\C=C\C(=O)C2=CC=C3C=CC=CC3=C2)C(Br)=C1	6,5	µM	IC50	MCF-7
**451.**	[H]C1CC[C@]2([H])[C@]1([H])CC[C@]1([H])C3=C(CC[C@@]21[H])C=C1OC(=CC(=O)C1=C3)C1=CC=CC=C1	5,6	µM	IC50	MCF-7
**452.**	[H]C1CC[C@]2([H])[C@]1([H])CC[C@]1([H])C3=C(CC[C@@]21[H])C=C1OC(=CC(=O)C1=C3)C1=CC=C(C)C=C1	8	µM	IC50	MCF-7

**453.**	[H]C1CC[C@]2([H])[C@]1([H])CC[C@]1([H])C3=C(CC[C@@]21[H])C=C1OC(=CC(=O)C1=C3)C1=CC=C(Br)C=C1	7,8	µM	IC50	MCF-7
**454.**	CON1C=C(C(=O)\C=C\C2=C(OC)C=C(OC)C=C2OC)C2=CC=CC=C12	< 7	µM	IC50	MCF-7
**455.**	CC1=NC(C)=C(C)N=C1\C=C\C(=O)C1=CC=CO1	5,07	µM	IC50	MCF-7
**456.**	COC1=CC=C2OC(=CC2=C1)C(=O)\C=C\C1=C(C)N=C(C)C(C)=N1	7,81	µM	IC50	MCF-7
**457.**	CC1=CSC(=C1)C1=CC=C(C=C1)C(=O)\C=C\C1=NC(C)=C(C)N=C1C	9,27	µM	IC50	MCF-7
**458.**	COC1=C(C=CC=C1)C(=O)\C=C\C1=CC=C(C=C1)[N+]([O-])=O	1,33	µM	GI50	MCF-7
**459.**	NC1CCCCC1CC1=CC(\C=C\C(=O)C2=CC=C(CC3=CC(=NC4=CC=CC=C34)C(F)(F)F)C=C2)=CC=C1	0,5	µM	GI50	MCF-7
**460.**	COC1=CC(OC)=C(C(=O)\C=C\C2=CC=C(C=C2)N2CCNCC2)C(O)=C1	3.08 a 16.13	%	percentual de inibição	MC-F7
**461.**	COC1=C(C(=O)\C=C\C2=CC=CC=C2)C(O)=C(C)C(O)=C1C	24	µM	IC50	MC-F7
**462.**	C1C(NN=C1C1=C2C=CC=CC2=CC2=C1C=CC=C2)C1=CC=CC=C1	0,73	µM	IC50	MC-F7
**463.**	ClC1=CC=C(C=C1)C1CC(=NN1)C1=C2C=CC=CC2=CC2=C1C=CC=C2	0,495	µM	IC50	MC-F7
**464.**	FC1=CC=C(C=C1)C1CC(=NN1)C1=C2C=CC=CC2=CC2=C1C=CC=C2	3,08	µM	IC50	MC-F7
**465.**	CC1=CC=C(C=C1)C1CC(=NN1)C1=C2C=CC=CC2=CC2=C1C=CC=C2	0,493	µM	IC50	MC-F7
**466.**	ClC1=CC(=CC=C1)C1CC(=NN1)C1=C2C=CC=CC2=CC2=C1C=CC=C2	0,66	µM	IC50	MC-F7
**467.**	[O-][N+](=O)C1=CC=C(C=C1)C1CC(=NN1)C1=C2C=CC=CC2=CC2=C1C=CC=C2	1,5	µM	IC50	MC-F7
**468.**	BrC1=CC=C(C=C1)C1CC(=NN1)C1=C2C=CC=CC2=CC2=C1C=CC=C2	1,5	µM	IC50	MC-F7
**469.**	FC1=CC(=CC=C1)C1CC(=NN1)C1=C2C=CC=CC2=CC2=C1C=CC=C2	2	µM	IC50	MC-F7
**470.**	[O-][N+](=O)C1=CC(=CC=C1)C1CC(=NN1)C1=C2C=CC=CC2=CC2=C1C=CC=C2	26	µM	IC50	MC-F7
**471.**	CN(C)C1=CC=C(C=C1)C1CC(=NN1)C1=C2C=CC=CC2=CC2=C1C=CC=C2	17	µM	IC50	MC-F7
**472.**	C1C(NN=C1C1=C2C=CC=CC2=CC2=C1C=CC=C2)C1=CC=C(C=C1)C1=CC=CO1	1,98	µM	IC50	MC-F7
**473.**	C1C(NN=C1C1=C2C=CC=CC2=CC2=C1C=CC=C2)C1=CC=CO1	23	µM	IC50	MC-F7

**474.**	COC1=CC(=CC(OC)=C1OC)C1CC(=NN1)C1=C2C=CC=CC2=CC2=C1C=CC=C2	29	µM	IC50	MC-F7
**475.**	COC1=C(OC)C=C(C=C1)C1CC(=NN1)C1=C2C=CC=CC2=CC2=C1C=CC=C2	25	µM	IC50	MC-F7
**476.**	COC1=CC=C(C=C1)C1CC(=NN1)C1=C2C=CC=CC2=CC2=C1C=CC=C2	2,5	µM	IC50	MC-F7
**477.**	C1C(NN=C1C1=C2C=CC=CC2=CC2=C1C=CC=C2)C1=CC=NC=C1	50	µM	IC50	MC-F7
**478.**	COC1=CC(OC)=C(C=C1S(N)(=O)=O)C(=O)\C=C\C1=CC=CC=C1	0,34	µM	IC50	MCF7
**479.**	COC1=CC(\C=C\C(=O)C2=CC=C(NC(=O)C3=CC=CC=C3CN3C(=O)C4=C(C=CC=C4)C3=O)C=C2)=CC(OC)=C1OC	1,88	µM	IC50	MCF-7
**480.**	COC1=CC(\C=C\C(=O)C2=CC=C3OC(=O)N(CN4CCCCC4)C3=C2)=CC(OC)=C1OC	14,6	µM	IC50	MDA-B-231
**481.**	COC1=CC(\C=C\C(=O)C2=CC=C3OC(=O)N(CN4CCOCC4)C3=C2)=CC(OC)=C1OC	19,6	µM	IC50	MDA-B-231
**482.**	COC1=CC(\C=C\C(=O)C2=CC=C3OC(=O)N(CN4CCSCC4)C3=C2)=CC(OC)=C1OC	7,4	µM	IC50	MDA-B-231
**483.**	COC1=CC(\C=C\C(=O)C2=CC=C3OC(=O)N(CN4CCN(CC5=CC=CC=C5)CC4)C3=C2)=CC(OC)=C1OC	12,7	µM	IC50	MDA-B-231
**484.**	COC1=CC(\C=C\C(=O)C2=CC=C3OC(=O)N(CN4CCN(CC4)C4=C(OC)C=CC=C4)C3=C2)=CC(OC)=C1OC	10,7	µM	IC50	MDA-B-231
**485.**	COC1=CC(OC)=C(C(=O)\C=C\C2=CC=C(C=C2)N2CCNCC2)C(O)=C1	4.99 a 35.58	%	percentual de inibição	MDA-MB-231
**486.**	CCOC1=CC=C(C=C1)C(=O)\C=C\C1=CC=C(C=C1)N(O)O	53,47	µM	IC50	MDA-MB-231
**487.**	CCOC1=CC=C(C=C1)C(=O)\C=C\C1=CC=C(Cl)C=C1	62,42	µM	IC50	MDA-MB-231
**488.**	CCOC1=CC=C(C=C1)C(=O)\C=C\C1=CC=C(Br)C=C1	54,04	µM	IC50	MDA-MB-231
**489.**	CCOC1=CC=C(C=C1)C(=O)\C=C\C1=CC=C(OC)C=C1	55.96 e	µM	IC50	MDA-MB-231

**490.**	CCOC1=CC=C(C=C1)C(=O)\C=C\C1=CC=C(OC)C(OC)=C1	57,3	μM	IC50	MDA-MB-231
**491.**	COC1=CC=C(C=C1)C1=CC2=CC=CC=C2N=C1\C=C\C(=O)C1=CC(OC)=C(OC)C(OC)=C1	0,75	μM	IC50	MDA-MDB-231
**492.**	COC1=CC(OC)=C(C=C1S(N)(=O)=O)C(=O)\C=C\C1=CC=CC=C1	0,34	μM	IC50	MDA-MB-468
**493.**	COC1=CC(OC)=C(C=C1S(N)(=O)=O)C(=O)\C=C\C1=CC=C(C)C=C1	1,96	μM	IC50	MDA-MB-468
**494.**	COC1=CC=C(\C=C\C(=O)C2=CC(OC)=C(OC)C(OC)=C2)C=C1OC(=O)CCC(=O)O[Pt]([NH3])([NH3])(O)(Cl)Cl	0,3	μM	IC50	MGC-803
**495.**	COC1=CC=C(\C=C\C(=O)C2=CC(OC)=C(OC)C(OC)=C2)C=C1OC(=O)CCC(=O)O[Pt]([NH3])([NH3])(Cl)(Cl)Cl	0,45	μM	IC50	MGC-803
**496.**	COC1=CC=C(\C=C\C(=O)C2=CC(OC)=C(OC)C(OC)=C2)C=C1OC(=O)CCCC(=O)O[Pt]([NH3])([NH3])(O)(Cl)Cl	0,26	μM	IC50	MGC-803
**497.**	COC1=CC=C(\C=C\C(=O)C2=CC(OC)=C(OC)C(OC)=C2)C=C1OC(=O)CCCC(=O)O[Pt]([NH3])([NH3])(Cl)(Cl)Cl	0,39	μM	IC50	MGC-803
**498.**	[H]C1=C(OC)C(OC)=CC=C1\C=C\C(=C)C1=C(C)N(N=N1)C1=CC=C(Br)C=C1	96,87	μM	IC50	MOLT-4
**499.**	[H]C1=C([H])C(\C=C\C(=O)C2=C(C)N(N=N2)C2=CC=C(Br)C=C2)=CC=C1OC	78,38	μM	IC50	MOLT-4
**500.**	[H]C1=CC=C(\C=C\C(=O)C2=C(C)N(N=N2)C2=CC=C(Br)C=C2)C(OC)=C1[H]	101,85	μM	IC50	MOLT-4
**501.**	[H]C1=CC=C(\C=C\C(=O)C2=C(C)N(N=N2)C2=CC=C(Br)C=C2)C([H])=C1[N+]([O-])=O	92,64	μM	IC50	MOLT-4
**502.**	[H]C1=C([H])C(\C=C\C(=O)C2=C(C)N(N=N2)C2=CC=C(Br)C=C2)=CC=C1Cl	95,46	μM	IC50	MOLT-4
**503.**	[H]C1=C([H])C(=CC=C1\C=C\C(=O)C1=C(C)N(N=N1)C1=CC=C(F)C=C1)N(C)C	102,53	μM	IC50	MOLT-4

**504.**	[H]C1=C(OC)C(OC)=CC=C1\C=C\C(=O)C1=C(C)N(N=N1)C1=CC=C(F)C=C1	109,65	µM	IC50	MOLT-4
**505.**	[H]C1=C([H])C(\C=C\C(=O)C2=C(C)N(N=N2)C2=CC=C(F)C=C2)=CC=C1OC	81,73	µM	IC50	MOLT-4
**506.**	[H]C1=CC=C(\C=C\C(=O)C2=C(C)N(N=N2)C2=CC=C(F)C=C2)C(OC)=C1[H]	104,76	µM	IC50	MOLT-4
**507.**	[H]C1=C([H])C(=CC=C1\C=C\C(=O)C1=C(C)N(N=N1)C1=CC=C(C=C1)C(O)=O)N(C)C	44,99	µM	IC50	MOLT-4
**508.**	[H]C1=C(OC)C(OC)=CC=C1\C=C\C(=O)C1=C(C)N(N=N1)C1=CC=C(C=C1)C(O)=O	102,3	µM	IC50	MOLT-4
**509.**	[H]C1=C([H])C(\C=C\C(=O)C2=C(C)N(N=N2)C2=CC=C(C=C2)C(O)=O)=CC=C1OC	97,7	µM	IC50	MOLT-4
**510.**	[H]C1=CC=C(\C=C\C(=O)C2=C(C)N(N=N2)C2=CC=C(C=C2)C(O)=O)C(OC)=C1[H]	94,4	µM	IC50	MOLT-4
**511.**	CC(C)(C)OC(=O)N1CCN(CC1)C(=S)SCC(=O)NC1=CC=C(C=C1)C(=O)\C=C\C1=CC=CC=C1F	0,87	µM	IC50	MOLT-4
**512.**	[H]C1CC[C@]2([H])[C@]1([H])CC[C@]1([H])C3=C(CC[C@@]21[H])C=C1OC(=CC(=O)C1=C3)C1=CC=CC=C1	10,5	µM	IC50	MRC-5
**513.**	[H]C1CC[C@]2([H])[C@]1([H])CC[C@]1([H])C3=C(CC[C@@]21[H])C=C1OC(=CC(=O)C1=C3)C1=CC=C(C)C=C1	9,5	µM	IC50	MRC-5
**514.**	[H]C1CC[C@]2([H])[C@]1([H])CC[C@]1([H])C3=C(CC[C@@]21[H])C=C1OC(=CC(=O)C1=C3)C1=CC=C(Br)C=C1	>10	µM	IC50	MRC-5
**515.**	[H]C1=C([H])C(=CC=C1\C=C\C(=C)C1=C(C)N(N=N1)C1=CC=C(Br)C=C1)N(C)C	92,23	µM	IC50	NCI-H226
**516.**	[H]C1=C(OC)C(OC)=CC=C1\C=C\C(=C)C1=C(C)N(N=N1)C1=CC=C(Br)C=C1	70,69	µM	IC50	NCI-H226
**517.**	[H]C1=C([H])C(\C=C\C(=O)C2=C(C)N(N=N2)C2=CC=C(Br)C=C2)=CC=C1OC	90,71	µM	IC50	NCI-H226

**518.**	[H]C1=CC=C(\C=C\C(=O)C2=C(C)N(N=N2)C2=CC=C(Br)C=C2)C(OC)=C1[H]	100,09	μM	IC50	NCI-H226
**519.**	[H]C1=CC=C(\C=C\C(=O)C2=C(C)N(N=N2)C2=CC=C(Br)C=C2)C([H])=C1[N+]([O-])=O	89,4	μM	IC50	NCI-H226
**520.**	[H]C1=C([H])C(\C=C\C(=O)C2=C(C)N(N=N2)C2=CC=C(Br)C=C2)=CC=C1Cl	91,03	μM	IC50	NCI-H226
**521.**	[H]C1=C([H])C(=CC=C1\C=C\C(=O)C1=C(C)N(N=N1)C1=CC=C(F)C=C1)N(C)C	90,12	μM	IC50	NCI-H226
**522.**	[H]C1=C(OC)C(OC)=CC=C1\C=C\C(=O)C1=C(C)N(N=N1)C1=CC=C(F)C=C1	87,38	μM	IC50	NCI-H226
**523.**	[H]C1=C([H])C(\C=C\C(=O)C2=C(C)N(N=N2)C2=CC=C(F)C=C2)=CC=C1OC	90,57	μM	IC50	NCI-H226
**524.**	[H]C1=CC=C(\C=C\C(=O)C2=C(C)N(N=N2)C2=CC=C(F)C=C2)C(OC)=C1[H]	53,06	μM	IC50	NCI-H226
**525.**	[H]C1=C([H])C(=CC=C1\C=C\C(=O)C1=C(C)N(N=N1)C1=CC=C(C=C1)C(O)=O)N(C)C	97,68	μM	IC50	NCI-H226
**526.**	[H]C1=C(OC)C(OC)=CC=C1\C=C\C(=O)C1=C(C)N(N=N1)C1=CC=C(C=C1)C(O)=O	100,07	μM	IC50	NCI-H226
**527.**	[H]C1=C([H])C(\C=C\C(=O)C2=C(C)N(N=N2)C2=CC=C(C=C2)C(O)=O)=CC=C1OC	104,58	μM	IC50	NCI-H226
**528.**	[H]C1=CC=C(\C=C\C(=O)C2=C(C)N(N=N2)C2=CC=C(C=C2)C(O)=O)C(OC)=C1[H]	98,49	μM	IC50	NCI-H226
**529.**	[H]C1=C([H])C(=CC=C1\C=C\C(=C)C1=C(C)N(N=N1)C1=CC=C(Br)C=C1)N(C)C	95,69	μM	IC50	NCI-H23
**530.**	[H]C1=C(OC)C(OC)=CC=C1\C=C\C(=C)C1=C(C)N(N=N1)C1=CC=C(Br)C=C1	59,9	μM	IC50	NCI-H23
**531.**	[H]C1=C([H])C(\C=C\C(=O)C2=C(C)N(N=N2)C2=CC=C(Br)C=C2)=CC=C1OC	95,63	μM	IC50	NCI-H23

**532.**	[H]C1=CC=C(\C=C\C(=O)C2=C(C)N(N=N2)C2=CC=C(Br)C=C2)C(OC)=C1[H]	79,4	µM	IC50	NCI-H23
**533.**	[H]C1=CC=C(\C=C\C(=O)C2=C(C)N(N=N2)C2=CC=C(Br)C=C2)C([H])=C1[N+]([O-])=O	87,61	µM	IC50	NCI-H23
**534.**	[H]C1=C([H])C(\C=C\C(=O)C2=C(C)N(N=N2)C2=CC=C(Br)C=C2)=CC=C1Cl	93,49	µM	IC50	NCI-H23
**535.**	[H]C1=C([H])C(=CC=C1\C=C\C(=O)C1=C(C)N(N=N1)C1=CC=C(F)C=C1)N(C)C	97,08	µM	IC50	NCI-H23
**536.**	[H]C1=C(OC)C(OC)=CC=C1\C=C\C(=O)C1=C(C)N(N=N1)C1=CC=C(F)C=C1	92,76	µM	IC50	NCI-H23
**537.**	[H]C1=C([H])C(\C=C\C(=O)C2=C(C)N(N=N2)C2=CC=C(F)C=C2)=CC=C1OC	88,15	µM	IC50	NCI-H23
**538.**	[H]C1=CC=C(\C=C\C(=O)C2=C(C)N(N=N2)C2=CC=C(F)C=C2)C(OC)=C1[H]	42,93	µM	IC50	NCI-H23
**539.**	[H]C1=C([H])C(=CC=C1\C=C\C(=O)C1=C(C)N(N=N1)C1=CC=C(C=C1)C(O)=O)N(C)C	100,76	µM	IC50	NCI-H23
**540.**	[H]C1=C(OC)C(OC)=CC=C1\C=C\C(=O)C1=C(C)N(N=N1)C1=CC=C(C=C1)C(O)=O	104,58	µM	IC50	NCI-H23
**541.**	[H]C1=C([H])C(\C=C\C(=O)C2=C(C)N(N=N2)C2=CC=C(C=C2)C(O)=O)=CC=C1OC	98,49	µM	IC50	NCI-H23
**542.**	[H]C1=CC=C(\C=C\C(=O)C2=C(C)N(N=N2)C2=CC=C(C=C2)C(O)=O)C(OC)=C1[H]	87,58	µM	IC50	NCI-H23
**543.**	[H]C1=C([H])C(=CC=C1\C=C\C(=C)C1=C(C)N(N=N1)C1=CC=C(Br)C=C1)N(C)C	96,6	µM	IC50	NCI-H322M
**544.**	[H]C1=C(OC)C(OC)=CC=C1\C=C\C(=C)C1=C(C)N(N=N1)C1=CC=C(Br)C=C1	95,33	µM	IC50	NCI-H322M
**545.**	[H]C1=C([H])C(\C=C\C(=O)C2=C(C)N(N=N2)C2=CC=C(Br)C=C2)=CC=C1OC	92,63	µM	IC50	NCI-H322M

**546.**	[H]C1=CC=C(\C=C\C(=O)C2=C(C)N(N=N2)C2=CC=C(Br)C=C2)C(OC)=C1[H]	93,81	µM	IC50	NCI-H322M
**547.**	[H]C1=CC=C(\C=C\C(=O)C2=C(C)N(N=N2)C2=CC=C(Br)C=C2)C([H])=C1[N+]([O-])=O	99,01	µM	IC50	NCI-H322M
**548.**	[H]C1=C([H])C(\C=C\C(=O)C2=C(C)N(N=N2)C2=CC=C(Br)C=C2)=CC=C1Cl	104,46	µM	IC50	NCI-H322M
**549.**	[H]C1=C([H])C(=CC=C1\C=C\C(=O)C1=C(C)N(N=N1)C1=CC=C(F)C=C1)N(C)C	94,66	µM	IC50	NCI-H322M
**550.**	[H]C1=C(OC)C(OC)=CC=C1\C=C\C(=O)C1=C(C)N(N=N1)C1=CC=C(F)C=C1	90,81	µM	IC50	NCI-H322M
**551.**	[H]C1=C([H])C(\C=C\C(=O)C2=C(C)N(N=N2)C2=CC=C(F)C=C2)=CC=C1OC	70,6	µM	IC50	NCI-H322M
**552.**	[H]C1=CC=C(\C=C\C(=O)C2=C(C)N(N=N2)C2=CC=C(F)C=C2)C(OC)=C1[H]	96,63	µM	IC50	NCI-H322M
**553.**	[H]C1=C([H])C(=CC=C1\C=C\C(=O)C1=C(C)N(N=N1)C1=CC=C(C=C1)C(O)=O)N(C)C	91,34	µM	IC50	NCI-H322M
**554.**	[H]C1=C(OC)C(OC)=CC=C1\C=C\C(=O)C1=C(C)N(N=N1)C1=CC=C(C=C1)C(O)=O	108,57	µM	IC50	NCI-H322M
**555.**	[H]C1=C([H])C(\C=C\C(=O)C2=C(C)N(N=N2)C2=CC=C(C=C2)C(O)=O)=CC=C1OC	92,06	µM	IC50	NCI-H322M
**556.**	[H]C1=CC=C(\C=C\C(=O)C2=C(C)N(N=N2)C2=CC=C(C=C2)C(O)=O)C(OC)=C1[H]	98,34	µM	IC50	NCI-H322M
**557.**	[H]C1=C([H])C(=CC=C1\C=C\C(=C)C1=C(C)N(N=N1)C1=CC=C(Br)C=C1)N(C)C	100,35	µM	IC50	NCI-H460
**558.**	[H]C1=C(OC)C(OC)=CC=C1\C=C\C(=C)C1=C(C)N(N=N1)C1=CC=C(Br)C=C1	84,35	µM	IC50	NCI-H460
**559.**	[H]C1=C([H])C(\C=C\C(=O)C2=C(C)N(N=N2)C2=CC=C(Br)C=C2)=CC=C1OC	101,59	µM	IC50	NCI-H460

**560.**	[H]C1=CC=C(\C=C\C(=O)C2=C(C)N(N=N2)C2=CC=C(Br)C=C2)C(OC)=C1[H]	102,78	μM	IC50	NCI-H460
**561.**	[H]C1=CC=C(\C=C\C(=O)C2=C(C)N(N=N2)C2=CC=C(Br)C=C2)C([H])=C1[N+]([O-])=O	109,24	μM	IC50	NCI-H460
**562.**	[H]C1=C([H])C(\C=C\C(=O)C2=C(C)N(N=N2)C2=CC=C(Br)C=C2)=CC=C1Cl	92,45	μM	IC50	NCI-H460
**563.**	[H]C1=C([H])C(=CC=C1\C=C\C(=O)C1=C(C)N(N=N1)C1=CC=C(F)C=C1)N(C)C	96,61	μM	IC50	NCI-H460
**564.**	[H]C1=C(OC)C(OC)=CC=C1\C=C\C(=O)C1=C(C)N(N=N1)C1=CC=C(F)C=C1	28,43	μM	IC50	NCI-H460
**565.**	[H]C1=C([H])C(\C=C\C(=O)C2=C(C)N(N=N2)C2=CC=C(F)C=C2)=CC=C1OC	110,03	μM	IC50	NCI-H460
**566.**	[H]C1=CC=C(\C=C\C(=O)C2=C(C)N(N=N2)C2=CC=C(F)C=C2)C(OC)=C1[H]	107,26	μM	IC50	NCI-H460
**567.**	[H]C1=C([H])C(=CC=C1\C=C\C(=O)C1=C(C)N(N=N1)C1=CC=C(C=C1)C(O)=O)N(C)C	112,37	μM	IC50	NCI-H460
**568.**	[H]C1=C(OC)C(OC)=CC=C1\C=C\C(=O)C1=C(C)N(N=N1)C1=CC=C(C=C1)C(O)=O	96,14	μM	IC50	NCI-H460
**569.**	[H]C1=C([H])C(\C=C\C(=O)C2=C(C)N(N=N2)C2=CC=C(C=C2)C(O)=O)=CC=C1OC	101,71	μM	IC50	NCI-H460
**570.**	[H]C1=CC=C(\C=C\C(=O)C2=C(C)N(N=N2)C2=CC=C(C=C2)C(O)=O)C(OC)=C1[H]	28,43	μM	IC50	NCI-H460
**571.**	COC1=CC=C(\C=C\C(=O)C2=CC(OC)=C(OC)C(OC)=C2)C=C1OC(=O)CCC(=O)O[Pt]([NH3])([NH3])(O)(Cl)Cl	0,45	μM	IC50	NCI-H460
**572.**	COC1=CC=C(\C=C\C(=O)C2=CC(OC)=C(OC)C(OC)=C2)C=C1OC(=O)CCC(=O)O[Pt]([NH3])([NH3])(Cl)(Cl)Cl	0,61	μM	IC50	NCI-H460
**573.**	COC1=CC=C(\C=C\C(=O)C2=CC(OC)=C(OC)C(OC)=C2)C=C1OC(=O)CCCC(=O)O[Pt]([NH3])([NH3])(O)(Cl)Cl	0,37	μM	IC50	NCI-H460

**574.**	COC1=CC=C(\C=C\C(=O)C2=CC(OC)=C(OC)C(OC)=C2)C=C1OC(=O)CCCC(=O)O[Pt]([NH3])([NH3])(Cl)(Cl)Cl	0,55	μM	IC50	NCI-H460
**575.**	[H]C1=C([H])C(=CC=C1\C=C\C(=C)C1=C(C)N(N=N1)C1=CC=C(Br)C=C1)N(C)C	89,51	μM	IC50	NCI-H522
**576.**	[H]C1=C(OC)C(OC)=CC=C1\C=C\C(=C)C1=C(C)N(N=N1)C1=CC=C(Br)C=C1	67,73	μM	IC50	NCI-H522
**577.**	[H]C1=C([H])C(\C=C\C(=O)C2=C(C)N(N=N2)C2=CC=C(Br)C=C2)=CC=C1OC	87,91	μM	IC50	NCI-H522
**578.**	[H]C1=CC=C(\C=C\C(=O)C2=C(C)N(N=N2)C2=CC=C(Br)C=C2)C(OC)=C1[H]	84,85	μM	IC50	NCI-H522
**579.**	[H]C1=CC=C(\C=C\C(=O)C2=C(C)N(N=N2)C2=CC=C(Br)C=C2)C([H])=C1[N+]([O-])=O	86,35	μM	IC50	NCI-H522
**580.**	[H]C1=C([H])C(\C=C\C(=O)C2=C(C)N(N=N2)C2=CC=C(Br)C=C2)=CC=C1Cl	89,92	μM	IC50	NCI-H522
**581.**	[H]C1=C([H])C(=CC=C1\C=C\C(=O)C1=C(C)N(N=N1)C1=CC=C(F)C=C1)N(C)C	87,66	μM	IC50	NCI-H522
**582.**	[H]C1=C(OC)C(OC)=CC=C1\C=C\C(=O)C1=C(C)N(N=N1)C1=CC=C(F)C=C1	75,77	μM	IC50	NCI-H522
**583.**	[H]C1=C([H])C(\C=C\C(=O)C2=C(C)N(N=N2)C2=CC=C(F)C=C2)=CC=C1OC	84,37	μM	IC50	NCI-H522
**584.**	[H]C1=CC=C(\C=C\C(=O)C2=C(C)N(N=N2)C2=CC=C(F)C=C2)C(OC)=C1[H]	53,96	μM	IC50	NCI-H522
**585.**	[H]C1=C([H])C(=CC=C1\C=C\C(=O)C1=C(C)N(N=N1)C1=CC=C(C=C1)C(O)=O)N(C)C	92,91	μM	IC50	NCI-H522
**586.**	[H]C1=C(OC)C(OC)=CC=C1\C=C\C(=O)C1=C(C)N(N=N1)C1=CC=C(C=C1)C(O)=O	88,38	μM	IC50	NCI-H522
**587.**	[H]C1=C([H])C(\C=C\C(=O)C2=C(C)N(N=N2)C2=CC=C(C=C2)C(O)=O)=CC=C1OC	83,77	μM	IC50	NCI-H522

**588.**	[H]C1=CC=C(\C=C\C(=O)C2=C(C)N(N=N2)C2=CC=C(C=C2)C(O)=O)C(OC)=C1[H]	82,4	µM	IC50	NCI-H522
**589.**	COC1=CC(=CC(OC)=C1OC)C(=O)\C=C\C1=CNC2=CC=CC=C12	0,03	µM	IC50	PaCa-2
**590.**	COC1=CC(=CC(OC)=C1OC)C(=O)\C=C\C1=CN(C)C2=CC=CC=C12	0,091	µM	IC50	PaCa-2
**591.**	ClC1=CC=C(\C=C\C(=O)C2=CC=CC=C2NS(=O)(=O)C2=CC=CC=C2)C=C1	2,4	µM	IC50	PC-3
**592.**	[H]C1CC[C@]2([H])[C@]1([H])CC[C@]1([H])C3=C(CC[C@@]21[H])C=C1OC(=CC(=O)C1=C3)C1=CC=CC=C1	6,2	µM	IC50	PC-3
**593.**	[H]C1CC[C@]2([H])[C@]1([H])CC[C@]1([H])C3=C(CC[C@@]21[H])C=C1OC(=CC(=O)C1=C3)C1=CC=C(C)C=C1	8,7	µM	IC50	PC-3
**594.**	[H]C1CC[C@]2([H])[C@]1([H])CC[C@]1([H])C3=C(CC[C@@]21[H])C=C1OC(=CC(=O)C1=C3)C1=CC=C(Br)C=C1	9,5	µM	IC50	PC-3
**595.**	COC1=CC=C(\C=C\C(=O)C2=C(C)N(N=N2)C2=CC=C(Cl)C=C2)C=C1OC	0,64	µM	IC50	RPMI-8226
**596.**	[H]C1=C(OC)C(OC)=CC=C1\C=C\C(=C)C1=C(C)N(N=N1)C1=CC=C(Br)C=C1	67,93	µM	IC50	RPMI-8226
**597.**	[H]C1=C([H])C(\C=C\C(=O)C2=C(C)N(N=N2)C2=CC=C(Br)C=C2)=CC=C1OC	10,53	µM	IC50	RPMI-8226
**598.**	[H]C1=CC=C(\C=C\C(=O)C2=C(C)N(N=N2)C2=CC=C(Br)C=C2)C(OC)=C1[H]	93,81	µM	IC50	RPMI-8226
**599.**	[H]C1=CC=C(\C=C\C(=O)C2=C(C)N(N=N2)C2=CC=C(Br)C=C2)C([H])=C1[N+]([O-])=O	61,31	µM	IC50	RPMI-8226
**600.**	[H]C1=C([H])C(\C=C\C(=O)C2=C(C)N(N=N2)C2=CC=C(Br)C=C2)=CC=C1Cl	17,98	µM	IC50	RPMI-8226
**601.**	[H]C1=C([H])C(=CC=C1\C=C\C(=O)C1=C(C)N(N=N1)C1=CC=C(F)C=C1)N(C)C	87,57	µM	IC50	RPMI-8226
**602.**	[H]C1=C(OC)C(OC)=CC=C1\C=C\C(=O)C1=C(C)N(N=N1)C1=CC=C(F)C=C1	89,72	µM	IC50	RPMI-8226
**603.**	[H]C1=C([H])C(\C=C\C(=O)C2=C(C)N(N=N2)C2=CC=C(F)C=C2)=CC=C1OC	54,14	µM	IC50	RPMI-8226

**604.**	[H]C1=CC=C(\C=C\C(=O)C2=C(C)N(N=N2)C2=CC=C(F)C=C2)C(OC)=C1[H]	67,78	µM	IC50	RPMI-8226
**605.**	[H]C1=C([H])C(=CC=C1\C=C\C(=O)C1=C(C)N(N=N1)C1=CC=C(C=C1)C(O)=O)N(C)C	20,94	µM	IC50	RPMI-8226
**606.**	[H]C1=C(OC)C(OC)=CC=C1\C=C\C(=O)C1=C(C)N(N=N1)C1=CC=C(C=C1)C(O)=O	95,13	µM	IC50	RPMI-8226
**607.**	[H]C1=C([H])C(\C=C\C(=O)C2=C(C)N(N=N2)C2=CC=C(C=C2)C(O)=O)=CC=C1OC	99,41	µM	IC50	RPMI-8226
**608.**	[H]C1=CC=C(\C=C\C(=O)C2=C(C)N(N=N2)C2=CC=C(C=C2)C(O)=O)C(OC)=C1[H]	105,37	µM	IC50	RPMI-8226
**609.**	CCCCN1C(=O)C(\C=C\C(=O)\C=C\C2=CC3=C(C=CC=C3)N(CCCC)C2=O)=CC2=C1C=CC=C2	0,32	µM	GI50	RPMI-8226
**610.**	ClC1=CC=C(\C=C\C(=O)C2=CC=CC=C2NS(=O)(=O)C2=CC=CC=C2)C=C1	2,1	µM	IC50	SF-295
**611.**	COC1=CC=C(C=C1)C1=CC2=CC=CC=C2N=C1\C=C\C(=O)C1=CC=CC=C2	0,7	µM	IC50	SKBR-3
**612.**	COC1=CC=C(C=C1)C1=CC2=CC=CC=C2N=C1\C=C\C(=O)C1=CC(OC)=C(OC)C(OC)=C1	0,78	µM	IC50	SKBR-3
**613.**	C1C(NN=C1C1=C2C=CC=CC2=CC2=C1C=CC=C2)C1=CC=CC=C1	260	µM	IC50	SK-N-SH
**614.**	ClC1=CC=C(C=C1)C1CC(=NN1)C1=C2C=CC=CC2=CC2=C1C=CC=C2	290	µM	IC50	SK-N-SH
**615.**	FC1=CC=C(C=C1)C1CC(=NN1)C1=C2C=CC=CC2=CC2=C1C=CC=C2	254,22	µM	IC50	SK-N-SH
**616.**	CC1=CC=C(C=C1)C1CC(=NN1)C1=C2C=CC=CC2=CC2=C1C=CC=C2	258,42	µM	IC50	SK-N-SH
**617.**	ClC1=CC(=CC=C1)C1CC(=NN1)C1=C2C=CC=CC2=CC2=C1C=CC=C2	254,23	µM	IC50	SK-N-SH
**618.**	[O-][N+](=O)C1=CC=C(C=C1)C1CC(=NN1)C1=C2C=CC=CC2=CC2=C1C=CC=C2	250,66	µM	IC50	SK-N-SH
**619.**	BrC1=CC=C(C=C1)C1CC(=NN1)C1=C2C=CC=CC2=CC2=C1C=CC=C2	240,26	µM	IC50	SK-N-SH
**620.**	FC1=CC(=CC=C1)C1CC(=NN1)C1=C2C=CC=CC2=CC2=C1C=CC=C2	250,29	µM	IC50	SK-N-SH
**621.**	[O-][N+](=O)C1=CC(=CC=C1)C1CC(=NN1)C1=C2C=CC=CC2=CC2=C1C=CC=C2	3,55	µM	IC50	SK-N-SH
**622.**	CN(C)C1=CC=C(C=C1)C1CC(=NN1)C1=C2C=CC=CC2=CC2=C1C=CC=C2	260,22	µM	IC50	SK-N-SH

**623.**	C1C(NN=C1C1=C2C=CC=CC2=CC2=C1C=CC=C2)C1=CC=C(C=C1)C1=CC=CO1	1,88	µM	IC50	SK-N-SH
**624.**	C1C(NN=C1C1=C2C=CC=CC2=CC2=C1C=CC=C2)C1=CC=CO1	2	µM	IC50	SK-N-SH
**625.**	COC1=CC(=CC(OC)=C1OC)C1CC(=NN1)C1=C2C=CC=CC2=CC2=C1C=CC=C2	23,42	µM	IC50	SK-N-SH
**626.**	COC1=C(OC)C=C(C=C1)C1CC(=NN1)C1=C2C=CC=CC2=CC2=C1C=CC=C2	25,33	µM	IC50	SK-N-SH
**627.**	COC1=CC=C(C=C1)C1CC(=NN1)C1=C2C=CC=CC2=CC2=C1C=CC=C2	253,88	µM	IC50	SK-N-SH
**628.**	C1C(NN=C1C1=C2C=CC=CC2=CC2=C1C=CC=C2)C1=CC=NC=C1	58,33	µM	IC50	SK-N-SH
**629.**	COC1=C(O)C(C(=O)\C=C\C2=CC=C(O)C=C2)=C(OC)C(OC)=C1OC	56,41	µM	IC50	SK-OV-3
**630.**	COC1=CC(\C=C\C(=O)C2=C(OC)C(OC)=C(OC)C(OC)=C2O)=CC=C1O	32,53	µM	IC50	SK-OV-3
**631.**	COC1=CC=C(\C=C\C(=O)C2=C(OC)C(OC)=C(OC)C(OC)=C2O)C=C1O	1,6	µM	IC50	SK-OV-3
**632.**	COC1=C(O)C(O)=CC(\C=C\C(=O)C2=C(OC)C(OC)=C(OC)C(OC)=C2O)=C1	48,4	µM	IC50	SK-OV-3
**633.**	COC1=CC(\C=C\C(=O)C2=C(OC)C(OC)=C(OC)C(OC)=C2O)=CC(OC)=C1OC	10,53	µM	IC50	SK-OV-3
**634.**	COC1=CC(\C=C\C(=O)C2=CC=C3OC(=O)N(CN4CCCCC4)C3=C2)=CC(OC)=C1OC	3,6	µM	IC50	SKW-3
**635.**	COC1=CC(\C=C\C(=O)C2=CC=C3OC(=O)N(CN4CCOCC4)C3=C2)=CC(OC)=C1OC	7,1	µM	IC50	SKW-3
**636.**	COC1=CC(\C=C\C(=O)C2=CC=C3OC(=O)N(CN4CCSCC4)C3=C2)=CC(OC)=C1OC	5,4	µM	IC50	SKW-3
**637.**	COC1=CC(\C=C\C(=O)C2=CC=C3OC(=O)N(CN4CCN(CC5=CC=CC=C5)CC4)C3=C2)=CC(OC)=C1OC	3,7	µM	IC50	SKW-3
**638.**	COC1=CC(\C=C\C(=O)C2=CC=C3OC(=O)N(CN4CCN(CC4)C4=C(OC)C=CC=C4)C3=C2)=CC(OC)=C1OC	3,6	µM	IC50	SKW-3
**639.**	CC1=CC=C(C=C1)C(=O)\C=C\C1=CC=C(\C=N\NC(N)=N)C=C1	3,05	µM	IC50	SMMC-7721
**640.**	COC1=CC(OC)=C(C=C1S(N)(=O)=O)C(=O)\C=C\C1=CC=C(C)C=C1	1,84	µM	IC50	SNB-75
**641.**	CCCCN1C(=O)C(\C=C\C(=O)\C=C\C2=CC3=C(C=CC=C3)N(CCCC)C2=O)=CC2=C1C=CC=C2	0,32	µM	GI50	SR

**642.**	O=C(\C=C\C1=CN(N=C1C1=CC=CS1)C1=CC=CC=C1)C1=CC=CS1	56,9	%	% sobrevivência	T-47D
**643.**	CCC1=CC=C(\C=C\C(=O)C2=CC=C(C=C2)[N+]([O-])=O)C=C1	4,756	μM	IC50	tipo L
**644.**	OCC1=CC=C(\C=C\C(=O)C2=CC=C(OC(=O)CO[N+]([O-])=O)C=C2)C=C1CO	3,608	μM	IC50	tipo L
**645.**	CC1=CC(=CC(\C=C/C(=O)C2=CC=C(Cl)C=C2)=C1)C(=O)C1=CC=C(O[N+]([O-])=O)C=C1	5,211	μM	IC50	tipo L
**646.**	OC1=CC=C(C(=O)\C=C\C2=CC=C(O)C(O)=C2)C(OC2=CC=CC=C2)=C1	14,6	mM	IC50	TNF-α
**647.**	COC1=CC(\C=C\C(=O)C2=CC(OC)=C(OC)C=C2)=CC(OC)=C1OC	19,5	μM	IC50	U87
**648.**	CN(C)C1=CC=C(\C=C\C(=O)C2=CC=C(C)C=C2)C=C1	61,24	%	% sobrevivência	UACC-257
**649.**	CN(C)C1=CC=C(\C=C\C(=O)C2=CC=CS2)C=C1	67,64	%	% sobrevivência	UACC-257
**650.**	COC1=CC(OC)=C(C(=O)\C=C\C2=CC=CC=C2)C(O)=C1	25	Unidade	IC50	LoVo
**651.**	COC1=CC2=C(C(=O)CC(O2)C2=CC=CC=C2)C(OC)=C1	50	μM	IC50	LoVo
**652.**	CC1=NC(C)=C(C)N=C1\C=C\C(=O)C1=CC=CO1	7,9	μM	IC50	A375
**653.**	COC1=CC=C2OC(=CC2=C1)C(=O)\C=C\C1=C(C)N=C(C)C(C)=N1	5,16	μM	IC50	A375
**654.**	CC1=CSC(=C1)C1=CC=C(C=C1)C(=O)\C=C\C1=NC(C)=C(C)N=C1C	3,68	μM	IC50	A375
**655.**	CCOC1=CC=C(C=C1)C(=O)\C=C\C1=CC=C(Cl)C=C1	49,17	μM	IC50	A-375
**656.**	CCOC1=CC=C(C=C1)C(=O)\C=C\C1=CC=C(Br)C=C1	40,76	μM	ic50	A-375
**657.**	CCOC1=CC=C(C=C1)C(=O)\C=C\C1=CC=C(C=C1)N(O)O	76,01	μM	ic50	A-375
**658.**	CCOC1=CC=C(C=C1)C(=O)\C=C\C1=CC=C(OC)C=C1	71,19	μM	ic50	A-375
**659.**	CCOC1=CC=C(C=C1)C(=O)\C=C\C1=CC=C(OC)C(OC)=C1	47,06	μM	ic50	A-375
